# B Cells Can Trigger the T-Cell-Mediated Autoimmune Response Against Melanocytes in Psoriasis

**DOI:** 10.3390/cells14242002

**Published:** 2025-12-16

**Authors:** Mengwen He, Melissa Bernhardt, Akiko Arakawa, Song-Min Kim, Sigrid Vollmer, Burkard Summer, Yukiyasu Arakawa, Tatsushi Ishimoto, Andreas Schlosser, Jörg Christoph Prinz

**Affiliations:** 1 Department of Dermatology and Allergy, University Hospital, Ludwig-Maximilian-University of Munich, D-80337 Munich, Germany; hemwtj@126.com (M.H.); akikoxxx999@gmail.com (A.A.); chilbo.med@gmail.com (S.-M.K.); sigrid.vollmer@med.uni-muenchen.de (S.V.); burkhard.summer@med.uni-muenchen.de (B.S.); aara@koto.kpu-m.ac.jp (Y.A.); kochinootokotatihe@gmail.com (T.I.); 2 Rudolf Virchow Center, Center for Integrative and Translational Bioimaging, University Würzburg, D-97078 Würzburg, Germany; melissa.bernhardt@uni-wuerzburg.de (M.B.); andreas.schlosser@uni-wuerzburg.de (A.S.); 3 Department for Dermatology, Venereology and Allergology, Frankfurt University Hospital, D-60590 Frankfurt, Germany

**Keywords:** Psoriasis vulgaris, streptococcal tonsillopharyngitis, HLA-C*06:02, psoriasis trigger, B-cell immunopeptidome, autoimmune disease, melanocytes, crossreactive CD8^+^ T cells

## Abstract

**Highlights:**

**What are the main findings?**

- We find that under inflammatory conditions, B cells from streptococci-infected tonsils and blood of HLA-C*06:02-positive psoriasis patients activate a melanocyte-reactive T-cell receptor from a pathogenic psoriatic CD8^+^ T cell clone and also have autostimulatory properties for CD8^+^ T cells.
- We identify several self-peptides from the complex HLA-C*06:02 immunopeptidomes of B cells that may serve as autoantigens to activate the lesional psoriatic autoimmune response against melanocytes due to T-cell receptor polyspecificity.

**What is the implication of the main finding?**

- The proinflammatory environment of streptococcal tonsillopharyngitis, as an important trigger for psoriasis, might break the tolerance of pathogenic psoriatic CD8^+^ T cells to autoantigens presented by HLA-C*06:02 on B cells that subsequently react against melanocytes, inducing psoriasis.
- The findings open up potential avenues for the development of therapies targeting the pathogenic B-T cell interaction in psoriasis.

**Abstract:**

Psoriasis vulgaris is a T-cell-mediated skin disease that may involve an autoimmune response against melanocytes. It develops through still unexplained pathomechanisms. Streptococcal tonsillopharyngitis is a major trigger of psoriasis onset and relapses. HLA-C*06:02 is the main psoriasis risk gene. Here we find that B cells isolated from streptococci-infected tonsils or peripheral blood of HLA-C*06:02^+^ psoriasis patients stimulate an HLA-C*06:02-restricted melanocyte-reactive Vα3S1/Vβ13S1 T-cell receptor (TCR) from a lesional psoriatic CD8^+^ T cell clone in an IFN-γ-enhanced manner. Patients’ B cells furthermore induce proliferation of autologous blood CD8^+^ T cells. We identify several HLA-C*06:02-presented self-peptides in the immunopeptidomes we had isolated from four HLA-C*06:02 homozygous B-cell lines that stimulate the Vα3S1/Vβ13S1 TCR and differ from the melanocyte autoantigen recognized by this TCR. These data suggest that the proinflammatory environment of streptococcal tonsillopharyngitis may enable B cells to activate autoreactive CD8^+^ T cells that, owing to the polyspecificity of T-cell receptors, recognize several B-cell self-peptides presented by HLA-C*06:02 and subsequently cross-react against melanocytes in the skin, thereby triggering psoriasis. The capacity of B cells to stimulate a cross-reactive autoimmune response through HLA class I-presented B-cell peptides is a previously unknown mechanism in the induction of autoimmunity that could explain psoriasis onset and persistence.

## 1. Introduction

Psoriasis is a prevalent T-cell-mediated autoimmune skin disease affecting an estimated 125 million people worldwide (https://www.psoriasis.org/psoriasis-statistics/, last accessed on 14 November 2025). It evolves from a multifaceted genetic background [1,2], with HLA-C*06:02 standing out as phenotype-specific risk gene [3,4]. Psoriatic inflammation arises from the infiltration, activation, and clonal expansion of CD8^+^ T cells within the epidermis [5,6,7], driving psoriatic skin pathology through a T_c_17 cytokine profile [8,9,10,11,12]. Using a Vα3S1/Vβ13S1 T-cell receptor (TCR) from an epidermal CD8^+^ T-cell clone originating from the psoriatic plaque of an HLA-C*06:02^+^ patient [7], our previous work established that HLA-C*06:02 mediates an autoimmune response of CD8^+^ T cells against melanocytes, and we identified a peptide from ADAMTS-like protein 5 (ADAMTSL5) as a melanocyte autoantigen [13,14]. According to the crystal structure of the trimolecular complex composed of TCR, HLA molecule and autoantigen, the Vα3S1/Vβ13S1 TCR engages in a unique charge network with the HLA-C*06:02 α1-helix and exposed arginine residues from the ADAMTSL5 peptide [15].

Contrary to congenital ailments, psoriasis develops over the course of a lifetime, with a maximum incidence in the second or third decade of life [16]. Streptococcal tonsillopharyngitis frequently acts as a trigger of psoriasis onset and can cause exacerbation of chronic plaque psoriasis, especially in HLA-C*06:02-positive individuals [17,18,19]. It was therefore considered an event that could be used to explore the mechanisms underlying the development of psoriasis. Streptococcal superantigens or molecular mimicry between streptococcal M proteins and organ-specific proteins have been implicated in the activation of pathogenic psoriatic T cells [20,21,22], but direct evidence for this is lacking. The exact pathomechanisms inducing the psoriatic autoimmune response in predisposed individuals are thus still incompletely understood.

The identification of lesional psoriatic T-cell clones within the skin-homing fraction of tonsillar T cells in streptococci-induced psoriasis, along with psoriasis remission following tonsillectomy [17,23], suggests that T cells may link streptococcal angina and psoriatic inflammation. We therefore utilized the psoriatic Vα3S1/Vβ13S1 TCR expressed in a mouse T hybridoma reporter cell line with superfolder green fluorescent protein (sGFP) induction as a readout for TCR ligation [13] in an unbiased approach to search for potential triggers of the psoriatic autoimmune response in tonsil tissue of streptococcal tonsillopharyngitis.

Screening tonsillar cells from HLA-C*06:02^+^ patients tonsillectomized due to streptococcal-induced psoriasis, we unexpectedly discovered that B cells stimulate the melanocyte-reactive Vα3S1/Vβ13S1 TCR. In the immunopeptidomes that we isolated from HLA-C*06:02-homozygous B-cell lines (BCLs), we identified several HLA-C*06:02-presented B-cell self-peptides ligating the Vα3S1/Vβ13S1 TCR. These findings indicate that in the pro-inflammatory environment of streptococcal tonsillopharyngitis, B cells might prime autoreactive CD8^+^ T cells through HLA-C*06:02-presented self-peptides, which subsequently cross-react against melanocytes in the skin, initiating psoriasis. Previous studies had suggested that B cells may contribute to psoriasis pathogenesis by a cytokine imbalance with an elevated production of pro-inflammatory IL-6 and impaired production of anti-inflammatory IL-10 [24,25,26]. Increased numbers of activated B-cell subpopulations and plasma cells in blood and skin lesions of psoriasis patients were furthermore interpreted as evidence of a pathogenic role for B cells in psoriasis, although this role remained unclear [27,28]. Our findings now uncover a previously unrecognized pathomechanism in which B cells in a secondary lymphoid organ may stimulate autoreactive CD8^+^ T cells in an HLA class I-restricted manner, triggering autoimmune pathology against a distinct cell type in a distant organ.

## 2. Materials and Methods

### 2.1. Ethics Statements

The study conformed to the ethical principles for medical research involving human participants outlined in the Declaration of Helsinki and was approved by the Ethics Committee of the Ludwig Maximilian University, Munich, Germany (Ref. No. 151-16, approved on 21 March 2016).

### 2.2. Study Participants

Patients with Type 1 chronic plaque psoriasis, alongside healthy donors who had no history of psoriasis or other inflammatory or autoimmune conditions, were enrolled from the psoriasis clinic or hospital staff. Information on age, gender and donated samples of participants can be found in Table S1. There were no statistically significant differences in age or gender between the two groups. Participation was voluntary. Informed consent was obtained from all subjects involved in the study.

### 2.3. Human Materials

Tonsils had been obtained from three HLA-C*06:02-positive psoriasis patients with a history of treatment-refractory psoriasis of 1.5, 6, or 10 years [23]. They had undergone tonsillectomy because psoriasis onset and subsequent flares had been closely related to recurrent episodes of streptococcal tonsillopharyngitis caused by Lancefield Group A β-hemolytic streptococci, as proven by bacterial cultures from tonsillar swabs. All patients experienced a lasting remission of psoriasis following tonsillectomy.

Isolation of PBMCs from venous blood using Ficoll density gradient centrifugation and preparation of single-cell suspensions from tonsil tissue by repeated mechanical fragmentation were done as described [23].

### 2.4. Cell Lines

The Vα3S1/Vβ13S1 TCR hybridoma was generated from a lesional psoriatic CD8^+^ T-cell clone and maintained in culture as previously described [7,13]. The origins and culture conditions of WM278, HaCaT, and A431 cell lines were detailed in a prior study [13]. NCI-H1975, MCF7, KHOS, and HEK293FT cell lines were cultured in DMEM supplemented with 10% FCS, 1 mM sodium pyruvate, 1× MEM nonessential amino acids, 10 μg/mL ciprofloxacin, 100 U/mL penicillin, and 100 μg/mL streptomycin. The HLA class I-deficient cell line 721.221 (ATCC=CRL-1855) was transfected with full-length HLA-C*06:02 cloned into the pcDNA3.1 vector [13] and maintained in RPMI 1640 medium containing 10% FCS, 100 U/mL penicillin, 100 μg/mL streptomycin, 1 mM sodium pyruvate, 1× MEM non-essential amino acids, 10 μg/mL ciprofloxacin, and hygromycin B (500 μg/mL). The stably HLA-C*06:02-transfected C1R cell line was generously provided by Prof. Dr. Stefan Stevanović, Tübingen, Germany.

Epstein–Barr Virus (EBV)-transformed BCLs Fey, P16488, P17490, and PSO7 had formerly been generated in the lab, while D22 and e9453 were provided by the late Prof. Rudolf Wank, Immunotherapy Research Center, Munich, Germany. HLA-C*06:02 of samples and cell lines was determined by sequence-based typing at the Laboratory for Immunogenetics and Molecular Diagnostics, University of Munich, Germany, or by PCR restriction fragment length polymorphism analysis as described previously [13].

Six HLA-C*06:02-homozygous EBV-transformed BCLs (GM20771, GM11930, HG00131, HG00142, GM12286, and GM12046) were identified based on HLA typing data from the 1000 Genomes Project and obtained from the NIGMS Human Genetic Cell Repository and NHGRI Sample Repository for Human Genetic Research at the Coriell Institute for Medical Research (https://www.coriell.org/, Camden, NJ, USA, last accessed on 9 October 2025). Endoplasmic reticulum aminopeptidase 1 (ERAP1) haplotypes of the six BCLs were determined from the whole-genome data of the 1000 Genomes Project Phase 3 (IGSR Data Portal, https://www.internationalgenome.org/ last accessed on 9 October 2025) by combining 9 major SNP genotypes (rs3734016, rs26653, rs26618, rs27895, rs2287987, rs30187, rs10050860, rs17482078 and rs27044) [29], which were mapped to the amino acid residues of the canonical ERAP1 isoform (UniProt Q9NZ08). The ERAP1 haplotypes of these BCLs are listed in Table 1, and the complete HLA class I haplotypes are listed in Table S2.

### 2.5. Cell Separation and Cell Sorting

To separate or deplete T cells and B cells, tonsillar cell suspensions or PBMCs were suspended in PBS containing 2 mM EDTA and 0.5% FCS and incubated with CD3 or CD19 antibody-conjugated magnetic microbeads (20 μL/10^7^ cells, Miltenyi Biotec, Bergisch Gladbach, Germany) at 4 °C for 20 min and passed through an a priori equilibrated MACS column placed in the magnetic field of a MACS Separator (Miltenyi Biotec). Flowthrough and target cells eluted after demagnetization were pelleted via centrifugation, rinsed in RPMI 1640 medium, and resuspended in the appropriate medium for further use.

For fluorescence-activated cell sorting of target cell subsets (B cells, CD4^+^ T cells, CD8^+^ T cells, natural killer/NK cells, monocytes, plasmacytoid dendritic cells/pDCs), PBMCs were washed with PBS and incubated with fluorescence-conjugated antibody cocktails for 30 min at 4 °C. The stained cells were sorted using the FACSAria™ II Fusion cell sorter (BD Biosciences, Heidelberg, Germany) based on marker expression, achieving a >98% purity of sorted cell fractions. The antibody marker panels and gating strategies are given in Figure S2. All antibodies used are listed in Table S3.

### 2.6. Vα3S1/Vβ13S1-TCR Hybridoma Activation Assays

To identify potential target cells of the Vα3S1/Vβ13S1 TCR, triplicates of 1 × 10^4^/well Vα3S1/Vβ13S1-TCR hybridoma cells were co-cultured with each 2 × 10^4^ tonsil cells or PBMCs with or without the addition of IFN-γ, 100ng/mL (IFN-γ human recombinant, endotoxin-tested, Sigma Aldrich, St. Louis, MO, USA), 2 × 10^4^ primary cells of different origin, different cell lines transfected with HLA-C*06:02 (Table S4) or cells from EBV-transformed BCLs (Table S2) in 96-well plates. To identify autoantigenic self-peptides, Vα3S1/Vβ13S1-TCR hybridoma cells were co-cultured with WM278 cells or HLA-C*06:02-721.221 cells transfected with plasmid-encoded peptides or loaded with synthetic peptides as described below. After 24 hrs of co-culture, activation of the Vα3S1/Vβ13S1-TCR hybridoma was determined by analyzing sGFP-positive hybridoma cells using flow cytometry (see below). To inhibit Vα3S1/Vβ13S1-TCR hybridoma activation, a pan-HLA class I antibody (W6/32, Biolegend, San Diego, CA, USA) or mouse IgG2a isotype control (MOPC-173, Biolegend) was added at a final concentration of 10 µg/mL. Vα3S1/Vβ13S1-TCR hybridoma stimulation by plasmid-encoded peptides expressed in HLA-C*06:02^+^ WM278 cells was normalized to the basal activation by WM278 cells transfected with empty vector. Hybridoma stimulation induced by synthetic peptides presented by WM278 was normalized to hybridoma activation by WM278 cells loaded with synthetic FALK peptide, an HLA-C*06:02-presented peptide lacking antigenicity for the Vα3S1/Vβ13S1 TCR [13,30]. In the absence of IFN-γ, WM278 cells are not stimulatory for the Vα3S1/Vβ13S1 TCR [13,14]. The ADAMTSL5-nonamer served as a positive control in both TCR hybridoma stimulation experiments with plasmid-encoded or synthetic peptides.

**Figure 1:**
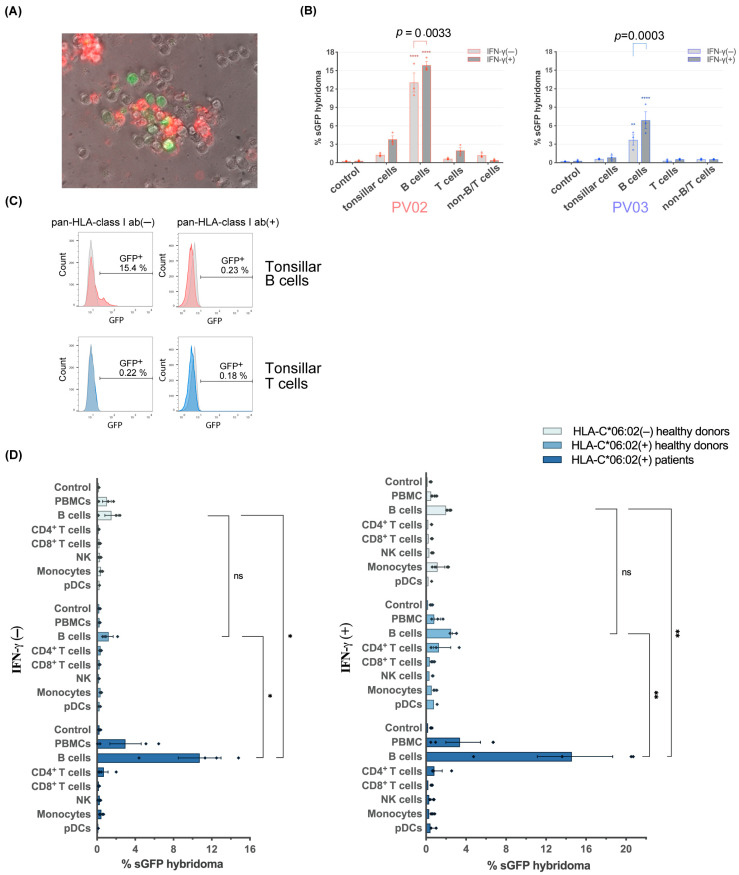
HLA-C*06:02^+^ tonsillar and peripheral blood B cells from psoriasis patients stimulate the melanocyte-reactive Vα3S1/Vβ13S1 TCR. (**A**) Vα3S1/Vβ13S1-TCR hybridoma cells induced to express sGFP (green) by co-culture with tonsil cells from an HLA-C*06:02^+^ psoriasis patient (PV1) stained with Alexa Fluor 647-labeled HLA-DR antibody (red), as shown in a merged UV light microscopy image. Unprocessed source photographs for the merged images are provided in Figure S1A. (**B**) Vα3S1/Vβ13S1-TCR activation by different tonsillar cell subsets from two HLA-C*06:02^+^ psoriasis patients (PV02 and PV03) without or with pre-incubation with IFN-γ compared to unstimulated hybridoma (control). Data are summarized from technical duplicates from three independent experiments and shown as mean ± SEM. Variation between the group means was analyzed using two-way ANOVA followed by Šídák-adjusted simple effects comparisons. Significance symbols denote multiplicity-adjusted two-tailed *p* values between stimulation by B cells *vs*. stimulation by each of the other cell subsets (i.e., control, tonsillar cells, T cells, and non-B/T cells) within each IFN-γ condition; **, *p* < 0.01; ****, *p* < 0.0001. The exact *p*-values above the bars indicate the significance of the differences in Vα3S1/Vβ13S1-TCR stimulation by B cells between IFN-γ (−) and IFN-γ (+) for PV02 and PV03. (**C**) Representative flow cytometry analysis of GFP^+^ hybridoma cells, with red histograms showing hybridoma stimulation by tonsillar B cells and blue histograms by tonsillar T cells in the absence (left) or presence (right) of the pan-HLA class I antibody W6/32; histograms of unstimulated hybridoma are shaded grey. (**D**) TCR hybridoma activation by co-culture with different PBMC subsets from HLA-C*06:02^−^ (light blue, *n* = 3, upper group) or HLA-C*06:02^+^ (blue, *n* = 3, middle group) healthy controls (HCs) or HLA-C*06:02^+^ psoriasis patients (dark blue, *n* = 4, lower group), without (left panel) or with (right panel) preincubation with IFN-γ. For the B-cell subset, group differences within each IFN-γ condition were analyzed using a two-way mixed-effects model (REML) followed by Šídák-adjusted simple effects comparisons. Data are shown as mean ± SEM of technical triplicates. *, *p* < 0.05; **, *p* < 0.01 (unpaired *t*-test). Unstimulated hybridoma cells served as negative control.

**Figure 2:**
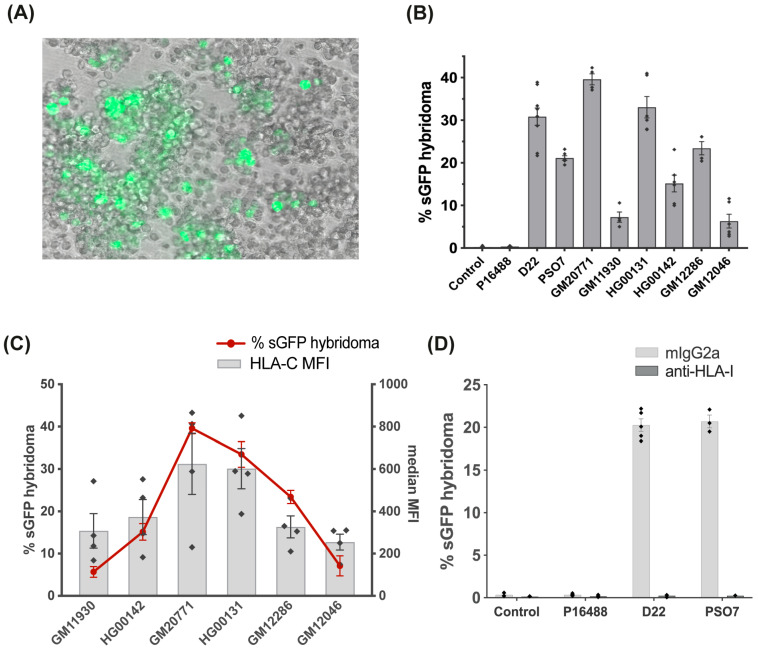
**Activation of the Vα3S1/Vβ13S1 TCR by various B-cell lines according to the HLA-C haplotype.** (**A**) Vα3S1/Vβ13S1 TCR hybridoma cells induced to express GFP (green) by co-culture with an HLA-C*06:02^+^ EBV-transformed BCL (merged UV light microscopy). Unprocessed source photographs for the merged images are provided in Figure S1B. (**B**) TCR hybridoma activation by various EBV-transformed HLA-C*06:02^+^ BCLs, but not the HLA-C*06:02^−^ BCL P16488. Table S1 lists the HLA class I haplotypes of each cell line. (**C)** Vα3S1/Vβ13S1 TCR hybridoma activation (red line, left axis; *n* = 4) by HLA-C*06:02-homozygous BCLs correlates with the expression level of HLA-C as determined by the mean fluorescence intensity (MFI) from DT-9 antibody staining and flow cytometry (grey bars with overlaid diamonds, right axis; *n* = 4). (**D**) Effect of the pan-HLA class I antibody W6/32 on the TCR hybridoma activation by HLA-C*06:02^+^ BCLs D22 and PSO7 and the HLA-C*06:02^-^ BCL P16488 (*n* = 3). Mouse IgG2a served as isotype control. Results in (**B**–**D**) represent the mean of triplicates from three or four independent experiments ± SEM, each depicted by a diamond. In (**B**,**D**), unstimulated hybridoma cells served as negative control.

For the analysis of co-culture-induced sGFP expression, cells were harvested and pelleted, and Vα3S1/Vβ13S1-TCR hybridoma cells were stained with PerCP/Cyanine5.5-conjugated CD8 antibody to differentiate them from other cell types (Figure S2A,B). In co-culture experiments with cell subsets expressing CD8, stimulatory cells were differentiated by staining with PerCP/Cyanine5.5-conjugated CD45 antibody, and sGFP expression in hybridoma cells was analyzed by flow cytometry using a FACSCanto™ II Flow Cytometry System (BD Biosciences). In each experiment, background activation and activability of the Vα3S1/Vβ13S1 TCR hybridoma were assessed by cultivating cells either untreated or in culture plates coated with CD3 antibodies for CD3 crosslinking (clone 17A2, eBioscience, San Diego, CA, USA) The maximum percentage of stimulable cells determined by CD3 cross-linking may reach 50 to 60% [31,32,33]. In antigen-specific stimulation, the percentage of hybridoma cells induced to express sGFP shows a direct correlation with the amount of peptide presented and is further influenced by the ratio between antigen-presenting cells and the TCR hybridoma cells, as shown in previous experiments that established or validated this technology [13,14,31,32,33]. Data were analyzed using Flow Jo software 8.8.7 (Tree Star, Ashland, OR, USA) by the recently published gating strategy [14]. Visual analysis of hybridoma activation was performed by fluorescence microscopy (AxioVert200M, Carl Zeiss Microscopy GmbH, Oberkochen, Germany; 520/35 BrightLine filter, Semrock, and 605/70 filter, IDEX Health & Science, Rochester, NY, USA). The unprocessed source photographs for the merged images in Figure 1A and Figure 2A are given in Figure S1.

### 2.7. Autologous Mixed Lymphocyte Reaction

To measure the stimulatory role of B cells in the autologous mixed lymphocyte reaction (AMLR), triplicates of 2 × 10^5^ PBMCs, B-cell-depleted PBMCs, or B-cell-reconstituted PBMCs reconstituted with autologous B cells were seeded in serum-free medium without an exogenous stimulus. Cells were pulsed on day four of culture overnight with 24,500 bq/well [^3^H]-thymidine (Hartmann Analytic, Braunschweig, Germany). Harvesting and measurement of radioactive decay were done on day 5 using an automatic filter counting system (Inotech Biosystems, Derwood, MD, USA). The results were expressed as mean of counts per minute (CPM) from triplicate measurements.

The effect of the pan-HLA class I antibody W6/32 on cell proliferation was measured by the BrdU Cell Proliferation Assay Kit (Cell Signaling Technology, Danvers, MA, USA) according to the manufacturer’s protocol. Briefly, triplicates of 2 × 10^5^ cells/well were seeded in 96-well plates and treated with a 10μM BrdU solution on day four of culture for a 4-h incubation. After staining with BrdU antibody and secondary HRP-linked antibody, HRP substrate TMB was added and color was quantified by measuring the absorbance at 450 nm in a Multiskan FC Photometer (ThermoFisher Scientific, Waltham, MA, USA).

Proliferation rates of PBMC subsets (CD8^+^ T cells, CD4^+^ T cells, CD3^+^ T cells, B cells) in the AMLR were measured in duplicates by a carboxyfluorescein diacetate succinimidyl ester (CFSE)-based assay, which determines the number of generations of a cell since the fluorescent label was applied. Between 1 × 10^7^ and 2 × 10^7^ PBMCs were incubated with CFSE (eBioscience) at a final concentration of 0.5 μM for 10 min at room temperature, washed three times and cultured for five days. Cells were stained with PE-CD3, PerCP/Cyanine5.5-CD8 and APC-CD19, or PE-CD8, PerCP/Cyanine5.5-CD4 and APC-CD19 antibodies (Table S3, Figure S2), and cellular CFSE was analyzed for each subset by flow cytometry. Figure S4 illustrates the workflow of the assays conducted to assess cell proliferation.

### 2.8. Analysis of HLA-C Expression

To determine the expression of HLA-C on BCLs, cells were stained with PE-labelled anti-human HLA-C (DT-9, BD Biosciences) or corresponding PE-labelled isotype control (Table S3) and then analyzed by a FACSCanto™ II flow cytometry system (BD). Data were analyzed by FlowJo (Tree Star) and expressed as mean fluorescence intensity (MFI).

### 2.9. Isolation of HLA Class I Ligands for Immunopeptidomics

Four HLA-C*06:02-homozygous B cell lines (GM20771, GM11930, HG00131, HG00142) were chosen for the isolation of HLA-C*06:02-associated peptides due to their stimulatory capacities for the Vα3S1/Vβ13S1 TCR. These cell lines were expanded in culture to each 1 × 10^9^ cells, harvested, washed three times in ice-cold PBS and stored as dry pellets at −80 °C until use. Isolation of HLA class I peptides was performed as described previously [34]. In brief, HG00313, HG00142, GM20771 and GM11930 cells were lysed in 1000 µL lysis buffer per 1 × 10^8^ cells. Immunopurification was performed with W6/32 antibody sepharose beads using the Waters Pressure Manifold. Peptide-MHC complexes were eluted with 1% trifluoroacetic acid and dried by lyophilization. HLA class I peptides were isolated by Restricted Access Material (RAM). RAM is based on a combination of size exclusion and reversed phase chromatography, enabling efficient separation of proteins and HLA peptides, as previously described [34]. For this purpose, freeze-dried eluates were resuspended in 200 μL of 10 mM ammonium acetate buffer, pH 7, and loaded on the RAM-Solid phase extraction column. The column was washed with 800 μL of 10 mM ammonium acetate buffer, pH 7, and peptides were eluted with 400 μL of 60% acetonitrile in 10 mM ammonium acetate buffer, pH 7. Finally, peptides were lyophilized and stored at −20 °C until LC-MS/MS analysis.

### 2.10. Analysis of HLA Class I Ligands by Liquid Chromatography-Tandem Mass Spectrometry (LC–MS/MS)

HLA peptides were resuspended in 30 µL 2% acetonitrile, 0.1% formic acid. LC-MS/MS was performed on an Orbitrap Fusion mass spectrometer coupled to an EASY-nLC 1000 (Thermo Scientific). MS method parameters are described in detail in a previous publication [34]. MS data were acquired in data-dependent mode (DDA). Raw data was processed with PEAKS X+ for de novo sequencing. Raw data refinement was conducted as described [34]. Peptide candidates obtained by de novo sequencing were matched against the 6-frame translated genome and the 3-frame translated human reference transcriptome with Peptide-PRISM 1.1.0. Results were filtered to 10% false discovery rate (FDR) (HLA_peptides_4_B_cell_lines_FDR_0.1.xlsx) or 1% FDR (HLA_peptides_4_B_cell_lines_FDR_0.01.xlsx) in a category-specific manner. With the more stringent filtering, the number of peptides decreased slightly. NetMHCpan 4.0 was used to calculate the %rank for each identified peptide for all given HLA class I-alleles of the corresponding cell line [35]. This “best binder analysis” defined strong binders (cut-off 0.5% rank) and weak binders (cut-off 2% rank) for HLA-C*06:02.

### 2.11. Definition of the Peptide Recognition Motif of the Vα3S1/Vβ13S1 TCR

The nonamer and octamer peptide recognition motifs of the Vα3S1/Vβ13S1 TCR (Figure S5A) were defined from previously identified peptide ligands. These include a total of 133 peptides identified by screening combinatorial peptide libraries, exchange of P2 and P9 against all previously defined HLA-C*06:02 anchor residues [30,36,37,38], alanine exchange of all amino acid residues, and by applying the continuously adapted peptide recognition motif to the human proteome and environmental proteins [13,39]. The heat map of preferred amino acids at different peptide positions and the corresponding Sequence Logo of the Vα3S1/Vβ13S1 TCR ligands corresponded to the HLA-C*06:02 peptide-binding motif [36,37,38] (Figure S5B). Q at position 5 was present in a stimulatory mimotope and in environmental peptide ligands [13,39]. The anchor residues of the ADAMTSL5 peptide (P2, P7 and P9) and the TCR contact residues (P5 and P8) were confirmed by crystal structure analysis of the Vα3S1/Vβ13S1 TCR–HLA-C*06:02 ADAMTSL5 complex [15,37].

### 2.12. Screening HLA-C*06:02-Immunopeptidomes with the Vα3S1/Vβ13S1 TCR Peptide Recognition Motif

To identify potential B-cell autoantigens of the Vα3S1/Vβ13S1 TCR, we initially screened two recently published HLA-C*06:02 immunopeptidomes isolated from the HLA-C*06:02-721.221 and C1R cell lines [37,38] with the nonamer and octamer recognition motifs of the Vα3S1/Vβ13S1 TCR (Figure S5A) for homologous peptides using the search algorithm shown in Figure S5C and selected 29 peptides for testing (Table S5). It combined anchor residues and TCR contact residues of previously identified Vα3S1/Vβ13S1-TCR ligands. When it became apparent during the experiments that HLA-C*06:02-721.221 and C1R cell lines did not stimulate the Vα3S1/Vβ13S1 TCR and therefore did not present Vα3S1/Vβ13S1 TCR ligands, we isolated the immunopeptidomes from four HLA-C*06:02-homozygous BCLs. Both predicted HLA-C*06:02 binders and non-binders were screened for potential Vα3S1/Vβ13S1 TCR ligands using the Vα3S1/Vβ13S1-TCR peptide recognition motif, and 81 peptides were selected for further testing (Table S6).

### 2.13. Cloning and Transfection of Candidate Peptides, Peptide Presentation

Cloning of cDNA of candidate peptides into pcDNA3.1D/V5-His-TOPO was done as previously described [13,39]. Nucleotide sequences of cloned peptides are provided in Tables S5 and S6. For peptide presentation, triplicates of WM278 cells or HLA-C*06:02-721.221 cells were seeded in 48-well plates or 96 well-plates at densities of 2.5 × 10^4^ or 2 × 10^4^ cells/well, respectively, and transfected with peptide-encoding plasmids (100 ng/well) using FuGENE HD reagent (Promega, Madison, WI, USA) or incubated with synthetic peptides (10 µg/mL), as described [13,39]. Highly purified synthetic candidate peptides (purity > 95%), synthetic ADAMTSL5 (VRSRRCLRL) and FALK peptide (VRHDGGNVL) were obtained from Thermo Fisher Scientific. Solubility of peptides was improved by adding 10% *v*/*v* dimethyl sulfoxide (DMSO).

### 2.14. Transcriptome Analysis of Cell Lines

Preparation and sequencing of barcoded RNA-seq libraries from total cellular RNA of cell lines HEK293FT, CaCo-2 and the BCL e9453 have been described previously [13]. Obtained 80-bp reads were mapped to the hg19 release of the human genome using spliced-read mapper Tophat and the UCSC gene annotation. Mapped reads overlapping with known genes were counted with HTseq count, available from the developers of DESeq.

### 2.15. Statistics and Reproducibility

Statistical analyses were performed in GraphPad Prism v10.5.0. All tests were two-tailed unless otherwise specified. Two-group comparisons used unpaired t tests or Mann–Whitney for nonparametric data; paired data used Wilcoxon signed-rank tests. Factorial designs used two-way ANOVA or mixed-effects (REML) with donor as a random intercept; significant interactions were probed by simple effects comparisons, with Šídák adjustment to control for multiple comparisons. Statistical significance was defined as adjusted *p* < 0.05; data are reported as mean ± SEM for approximately normal distributions. Sample sizes were informed by preliminary data and prior reports; exact *n* and the number of independent experiments are provided in figure legends.

## 3. Results

### 3.1. B Cells from Tonsils of HLA-C*06:02^+^ Patients with Streptococci-Driven Psoriasis Stimulate the Melanocyte-Specific Psoriatic Vα3S1/Vβ13S1 TCR

To identify potential triggers of the lesional psoriatic autoimmune response, we prepared single-cell suspensions from tonsils of three HLA-C*06:02^+^ psoriasis patients who had undergone tonsillectomy due to recurrent streptococci-driven psoriasis. Co-culturing these cell suspensions with the Vα3S1/Vβ13S1-TCR hybridoma revealed that tonsil cells effectively stimulated the Vα3S1/Vβ13S1 TCR, as evidenced by the induction of sGFP in the hybridoma cells (Figure 1A,B and Figure S1A).

To determine which cell population was responsible for TCR activation, we fractionated the tonsillar cell suspensions of two patients into B cells, T cells, and a “non-B/non-T cell” population. Only B cells stimulated the Vα3S1/Vβ13S1 TCR upon co-culture, whereas T cells and non-B/non-T cells failed to elicit a response (Figure 1B,C). Notably, pre-conditioning B cells with IFN-γ enhanced TCR hybridoma activation (Figure 1B). Conversely, hybridoma activation by B cells was inhibited by the pan-HLA class I antibody W6/32 (Figure 1C), indicating that B-cell-mediated stimulation of the Vα3S1/Vβ13S1 TCR was HLA class I-restricted.

### 3.2. Blood B Cells of HLA-C*06:02^+^ Psoriasis Patients Activate the Psoriatic Vα3S1/Vβ13S1-TCR Hybridoma

To assess the stimulatory capacity of B cells further, we separated peripheral blood mononuclear cells (PBMC) of HLA-C*06:02^-^ or HLA-C*06:02^+^ healthy individuals (each *n* = 3) or HLA-C*06:02^+^ psoriasis patients (*n* = 4) by fluorescence activated cell sorting into B cells, CD4^+^ or CD8^+^ T cells, natural killer (NK) cells, monocytes and plasmacytoid dendritic cells (PDCs) using the strategy shown in Figure S2. In co-culture experiments with these cell subsets, only HLA-C*06:02^+^ B cells elicited stimulation of the Vα3S1/Vβ13S1 TCR (Figure 1D). In particular, monocytes and plasmacytoid dendritic cells, which are professional antigen-presenting cells that primarily present exogenously ingested antigens, did not induce TCR hybridoma activation. Remarkably, B cells from HLA-C*06:02^+^ psoriasis patients triggered a significantly greater activation of the Vα3S1/Vβ13S1 TCR than those from HLA-C*06:02^+^ healthy individuals, which showed hardly any stimulating activity, and this effect was also enhanced by pre-incubation of the PBMC subsets with IFN-γ.

### 3.3. Stimulation of the Vα3S1/Vβ13S1-TCR Hybridoma by B Cells Is HLA-C*06:02-Restricted

To further determine the cell-type-specific reactivity of the Vα3S1/Vβ13S1 TCR and the HLA class I allele involved in its stimulation by B cells, we co-cultured the Vα3S1/Vβ13S1-TCR hybridoma with naturally HLA-C*06:02^+^ or HLA-C*06:02-transfected cells or cell lines of different origins. In addition to HLA-C*06:02^+^ primary B cells, only naturally HLA-C*06:02^+^ lymphoblastoid BCLs and an HLA-C*06:02^-^ BCL (P16488) transfected with HLA-C*06:02 activated the Vα3S1/Vβ13S1 TCR, whereas HLA-C*06:02^-^ BCLs and other HLA-C*06:02-transfected cell types did not (Figure 2A,B and Figure S3A–C and Table S4). Notably, skin-derived cell types other than melanocytes did not elicit Vα3S1/Vβ13S1-TCR stimulation: neither co-culture with HLA-C*06:02-transfected keratinocytes or the keratinocyte-derived cell lines A-431 and HaCaT nor genuinely HLA-C*06:02^+^ fibroblasts activated the TCR hybridoma (Table S4), further confirming that—in skin—the CD8^+^ T-cell clone from which the Vα3S1/Vβ13S1 TCR originates reacts specifically against melanocytes.

In stimulation experiments involving six different HLA-C*06:02-homozygous BCLs, the degree of hybridoma activation corresponded to the respective expression level of HLA-C*06:02 (Figure 2C), and this activation was completely blocked by the HLA class I antibody W6/32 (Figure 2D). Taken together, these results reveal that in addition to melanocytes, the Vα3S1/Vβ13S1 TCR selectively reacts against B cells in an HLA-C*06:02-restricted manner, presumably by recognizing B-cell autoantigens presented by HLA-C*06:02. The experiments further indicated that BCLs can replace primary B cells as unlimited cellular source in further experiments searching for B cell autoantigens.

### 3.4. B Cells Are Autostimulatory for CD8^+^ T Cells from Psoriasis Patients

**Figure 3:**
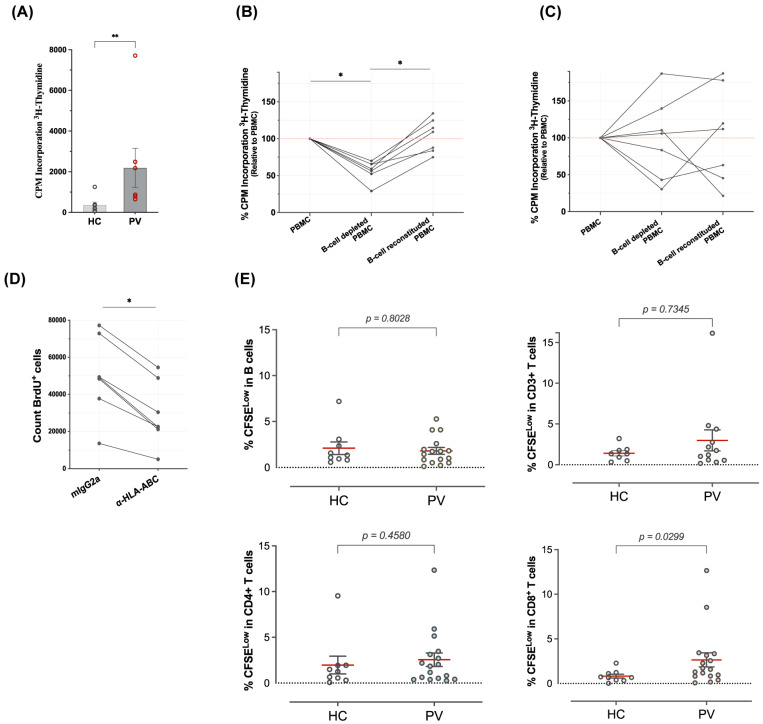
**B cells are autostimulatory for CD8^+^ T cells in psoriasis patients.** (**A**) Autoproliferation of PBMCs from HLA-C*06:02^+^ psoriasis patients (PVs) and healthy controls (HCs, each *n* = 7), measured in triplicates by [^3^H]-thymidine incorporation after 5 days of culture. Dots represent the mean values for each subject, and bars mark the mean ± SEM. **, *p* < 0.01 (Mann–Whitney U-test). (**B**) Spontaneous proliferation of B-cell-depleted and B-cell-reconstituted PBMCs from psoriasis patients (PV) and (**C**) healthy controls (HC; each *n* = 7) relative to autoproliferation of total PBMCs, as measured by incorporation of [^3^H]-Thymidine. (**D**) Inhibition of autoproliferation of the PBMCs of psoriasis patients by the pan-HLA class I-antibody W6/32 as compared to the isotype control, assessed by the decrease in BrdU^+^ cell counts. In (**B**–**D**), dots represent the mean values of triplicates, and lines represent the value changes in each subject; * *p* < 0.05 (Wilcoxon matched-pairs signed rank test). (**E**) Proportion of proliferating cells in different cell subsets of PBMCs (B cells, CD3^+^, CD4^+^ and CD8^+^ cells) of healthy controls and psoriasis patients (*n* = 9 HC *vs. n* = 17 PV), determined by the frequency of cells with decreased CFSE content (% CFSE^low^ cells) after 5 days of culture without addition of exogenous stimulators. Dots represent the mean values of duplicates for each subject, and bars mark the mean ± SEM of the group. *p* values were determined by two-tailed Mann–Whitney U-test.

Next, we investigated whether the reactivity against B cells represents an idiotypic property of the Vα3S1/Vβ13S1 TCR or indicates a general responsiveness of CD8^+^ T cells from psoriasis patients according to the workflow given in Figure S4. We cultured freshly seeded PBMCs in serum-free medium without additional stimulators and determined the proliferation by measuring [^3^H]-thymidine incorporation after 5 days. We observed that PBMCs from HLA-C*06:02^+^ psoriasis patients exhibited significantly higher autoproliferation (AP) compared to healthy individuals (each *n* = 7) (Figure 3A). AP of psoriasis PBMCs was diminished upon B-cell depletion and reinstated upon re-addition of B cells (Figure 3B). AP of PBMCs from HCs was not uniformly affected by depletion and re-addition of B cells (Figure 3C). AP of the psoriatic PBMCs was inhibited by the pan-HLA class I antibody W6/32 (Figure 3D), indicating HLA class I-restricted stimulation.

We then employed the dilution of carboxyfluorescein diacetate N-succinimidyl ester (CFSE) to differentiate proliferating from non-proliferating PBMC subsets in psoriasis patients (*n* = 17) and healthy controls (*n* = 9). After five days of culture with CFSE, we stained the cells with monoclonal antibodies for CD3, CD4, CD8 or CD19, and determined the rounds of cell division in each subset by FACS analysis. While the proliferative activity of B cells, CD3^+^ T cells or CD4^+^ T cells did not differ significantly between healthy subjects and psoriasis patients, the proliferation of CD8^+^ T cells was significantly higher in psoriasis patients than in healthy subjects (Figure 3E and Figure S4). Thus, in the autologous mixed lymphocyte reaction, B cells from psoriasis patients induced a marked autostimulation of CD8^+^ T cells in an HLA class I-restricted manner.

### 3.5. The HLA Class I-Deficient HLA-C*06:02-Transfected B-Cell Lines C1R and 721.221 Do Not Stimulate the Vα3S1/Vβ13S1 TCR

The HLA-C*06:02-restricted stimulation of the Vα3S1/Vβ13S1 TCR hybridoma by HLA-C*06:02^+^ B cells or BCLs and its blockade by a pan-HLA class I antibody is consistent with antigen-specific activation by self-peptides presented by HLA-C*06:02. In a first attempt to identify stimulatory peptides, we screened two recently published HLA-C*06:02 immunopeptidomes of two HLA class I-deficient and HLA-C*06:02-transfected BCLs, 721.221 and C1R [37,38] for peptides corresponding to the precisely characterized peptide recognition motif of the Vα3S1/Vβ13S1 TCR (Figure S5A,C). 721.221 has undergone a gamma-ray-induced homozygous deletion of the HLA class I region, resulting in the absence of endogenous HLA class I expression [40]. C1R shows a gamma-ray-induced deletion of the HLA-A3, Bw62, and Cw3 allele, and a point mutation in the translation initiation codon of the retained HLA-B35 allele [41,42]. The loss of the endogenous HLA class I expression of both cell lines allows for controlled reconstitution with specific HLA class I alleles and the analysis of the corresponding immunopeptidomes. None of the 29 candidate peptides (Table S5) that we had selected from the HLA-C*06:02 immunopeptidomes of these cell lines, cloned as minigenes and expressed for presentation in the HLA-C*06:02^+^ WM278 cell line, activated the Vα3S1/Vβ13S1 TCR. Subsequent co-culture experiments revealed that neither 721.221 nor C1R cells, despite their B-cell origins, activated the Vα3S1/Vβ13S1 TCR (Figure S3A,C). The inability of both HLA-C*06:02-transfected cell lines to stimulate the Vα3S1/Vβ13S1 TCR suggested an altered spectrum of HLA-C*06:02-presented antigens compared to unmodified HLA-C*06:02-positive BCLs. Thus, the immunopeptidomes of both cell lines were not suitable for the identification of Vα3S1/Vβ13S1-TCR ligands.

### 3.6. The HLA-C*06:02 Immunopeptidomes of Four HLA-C*06:02-Homzygous B-Cell Lines Are Complex

**Figure 4:**
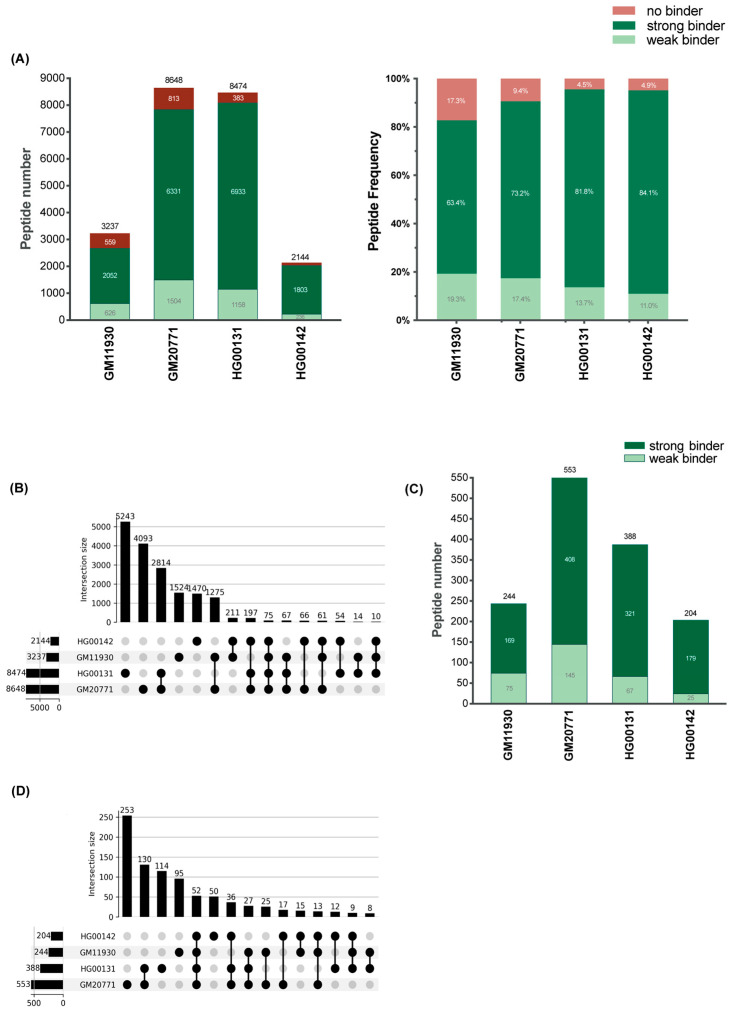
Characteristics of the immunopeptidomes from the four HLA-C*06:02-homozygous BCLs. (**A**) Total yield of peptides of each immunopeptidome, differentiated according to binding affinity to the respective HLA class I haplotypes of the four BCLs, given as number and percentage, as determined with NetMHCPan 4.0. (**B**) Overlap between the immunopeptidomes of the four BCLs, illustrated by an upset plot of shared and unique peptide groups. The vertical bar chart shows the total number of peptides shared between different cell lines, represented by filled dots. (**C**) Differentiation of the peptides of the four HLA-C*06:02 immunopeptidomes according to their binding affinity to HLA-C*06:02 (strong binder: cut-off 0.5% rank; weak binder: cut-off 2% rank, defined by NetMHCPan 4.0). (**D**) Upset plot depicting unique peptide groups and overlap between the HLA-C*06:02 immunopeptidomes from the four HLA-C*06:02-homzygous BCLs.

In a second approach to search for B-cell peptides that are antigenic for the Vα3S1/Vβ13S1 TCR, we analyzed the immunopeptidomes of four HLA-C*06:02 homozygous BCLs that elicited robust stimulation of the Vα3S1/Vβ13S1 TCR (GM11930, HG00142, GM20771, HG00131, Figure 2C). When filtered to 1% FDR, a total of 17,174 unique peptides were identified (HLA_peptides_4_B_cell_lines_FDR_0.1.xlsx) (https://doi.org/10.6084/m9.figshare.30608489). Two cell lines produced high HLA peptide yields (GM20771 & HG00131; 8474 or 8648 peptides), and two showed moderate yields (GM11930 & HG00142; 3237 or 2144 peptides, Figure 4A). Of these, the majority of peptides could be assigned to one of the HLA class I alleles expressed by the respective cell line in the analysis with NetMHCpan 4.0, while between 4.9% and 17.3% were categorised as non-binders that could not be mapped to any of the HLA class I alleles. Owing to the different HLA class I haplotypes, except for HLA-C*06:02 homozygosity (Table S2), the four immunopeptidomes showed only limited overlap (Figure 4B). It was greater between the two cell lines with high peptide yields and a shared HLA-A haplotype (HLA-A*01:01, HLA-A*03:01, Table S2).

In total, 856 peptides were classified as HLA-C*06:02 binders, the majority of which were strong binders (Figure 4C). The peptide yields of the individual BCLs corresponded to the respective expression levels of HLA-C*06:02 (Figure 2C). The cell lines GM20771 and HG00131, which exhibited the highest HLA-C*06:02 expression among the BCLs, provided a higher peptide yield and greater peptide overlap than GM11930 and HG00142, which showed a lower HLA-C*06:02 expression. 245/388 (63%) of HLA-C*06:02 peptides identified for HG00131 were shared with GM20771. 52 of the HLA-C*06:02 binders were detected in all BCLs (Figure 4D). In line with previous reports on HLA-C*06:02 immunopeptidomes [37,38,43], the majority of HLA-C*06:02 binders had a length of 9 amino acids, a minor fraction consisted of 8 amino acids, and only select peptides were 10 or 11 amino acids long (Table 1). The sequence motif of HLA-C*06:02 binders (Figure S5B) corresponded to the peptide motif of HLA-C*06:02 identified in earlier studies [36,37,38]. The ADAMTSL5 peptide, which acts as an autoantigen for melanocytes, was not observed in the four immunopeptidomes. This is consistent with the very low expression or absence of ADAMTSL5 in the B-cell transcriptome (https://doi.org/10.6084/m9.figshare.30814700) or proteome (https://www.proteinatlas.org/ENSG00000185761-ADAMTSL5/single+cell, accessed last 15 December 2025).

### 3.7. Various Self-Peptides from the HLA-C*06:02 Immunopeptidomes of HLA-C*06:02-Homozygous B-Cell Lines Are Autoantigens of the Vα3S1/Vβ13S1 TCR

To search for Vα3S1/Vβ13S1-TCR ligands other than the ADAMTSL5 peptide, we screened the four immunopeptidomes with the peptide screening motifs shown in Figure S5A,C and selected 81 peptides for further testing (Table S6). We cloned the cDNA corresponding to these peptides into plasmids and expressed them for presentation in the HLA-C*06:02^+^ melanoma cell line WM278. WM278 cells themselves only activate the Vα3S1/Vβ13S1 TCR when conditioned with IFN-γ, due to the inherent presentation of the ADAMTSL5 peptide. In the absence of IFN-γ, their antigenicity for the Vα3S1/Vβ13S1 TCR is negligible [13,14]. To compensate for any background stimulation, activation of the Vα3S1/Vβ13S1 TCR was normalized to the basal activation by WM278 cells transfected with empty vector, with the plasmid-encoded ADAMTSL5 nonamer serving as a positive control.

**Figure 5:**
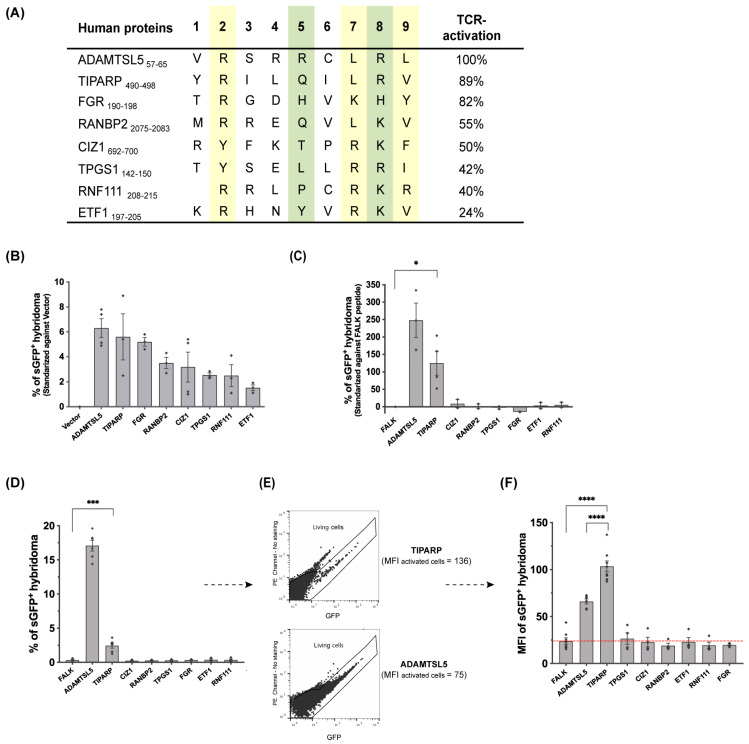
Various self-peptides from the HLA-C*06:02 immunopeptidomes of four HLA-C*06:02-homozygous B-cell lines stimulate the Vα3S1/Vβ13S1 TCR. (**A**,**B**) Plasmid-encoded peptides stimulating the Vα3S1/Vβ13S1 TCR identified through screening of HLA-C*06:02-B cell immunopeptidomes presented by WM278 cells. Stimulation was normalized to the basal activation by WM278 cell transfected with empty vector. The ADAMTSL5 peptide served as positive control. Percentages of TCR hybridoma stimulation in (**A**) represent the peptide-induced stimulation relative to the ADAMTSL5 peptide. Anchor residues for HLA-C*06:02 are highlighted in yellow, TCR contact residues in green. Amino acids are designated by the one-letter code. Vα3S1/Vβ13S1-TCR hybridoma activation by various synthetic peptides presented by WM278 (**C**) or HLA-C*06:02-721.221 cells (**D**). (**E**) Representative flow cytometry panels of Vα3S1/Vβ13S1-TCR hybridoma activation with synthetic TiPARP (upper panel) or ADAMTSL5 peptide (lower panel) and (**F**) Mean fluorescence intensity (MFI) of hybridoma cells stimulated by various synthetic peptides presented by HLA-C*06:02-721.221 cells. Data are summarized from technical triplicates from three or more independent experiments each represented by a diamond and shown as mean ± SEM. Differences were analyzed by two-way ANOVA with Dunnett correction versus the non-stimulatory FALK peptide; additionally, a pre-specified TiPARP *vs*. ADAMTSL5 comparison was tested with Šídák adjustment. Significance symbols denote multiplicity-adjusted two-tailed *p* values (*, *p* < 0.05; ***, *p* < 0.001; ****, *p* < 0.0001).

Seven of the 81 plasmid-encoded candidate peptides stimulated the Vα3S1/Vβ13S1-TCR hybridoma (Figure 5A,B), thus broadening the spectrum of peptides recognized by the Vα3S1/Vβ13S1 TCR due to TCR polyspecificity [13,39]. They included peptides derived from the proteins TCDD Inducible Poly (ADP-Ribose) Polymerase/Protein mono-ADP-ribosyltransferase (TIPARP, UniProt entry Q7Z3E1·PARPT_HUMAN), Tyrosine-protein kinase Fgr/Proto-Oncogene c-Fgr (FGR, P09769·FGR_HUMAN), Ran-binding protein 2 (RANBP2, P49792·RBP2_HUMAN), Cip1-interacting zinc finger protein 1 (CIZ1, Q9ULV3·CIZ1_HUMAN), Tubulin polyglutamylase complex subunit 1 (TPGS1, Q6ZTW0·TPGS1_HUMAN), E3 ubiquitin-protein ligase Arkadia/RING finger protein 111 (RNF111, Q6ZNA4·RN111_HUMAN), and Eukaryotic peptide chain release factor subunit 1 (ETF1, P62495·ERF1_HUMAN). The peptides were all strong binders to HLA-C*06:02 according to NetMHCpan 4.0 and the updated version, NetMHCPan 4.1 [44] (Table S7), except for the RNF111 peptide, which originated from the non-binder fraction (FDR threshold of 1,06% ≥ RRLPCRKR, q-value of 0.0106). Transcriptome analysis of a stimulatory EBV-transformed BCL, e9453, confirmed that all peptides originate from the B-cell proteome (https://doi.org/10.6084/m9.figshare.30814700).

Each B-cell immunopeptidome contained between one and three of the stimulating peptides (Table 1). The heterogeneity of the ligandomes and the uneven distribution of stimulating peptides among the four BCLs involve methodological aspects and are primarily attributable to the limited sensitivity and variance in the purification of HLA peptides [45]. However, different activities in peptide processing may also have an effect, especially as the four BCLs express different ERAP1 haplotypes with different enzymatic activity (Table 1), but these cannot be reliably determined in a qualitative comparison of ligandomes with limited immunopeptidome coverage.

### 3.8. The Antigenicity of the Parental Proteins of the Peptides Stimulating the Vα3S1/Vβ13S1 TCR Is Cell-Type Dependent

We then analysed the expression pattern of the parental proteins of the seven stimulatory peptides from the B-cell immunopeptidomes in the Human Protein Atlas (https://www.proteinatlas.org/) and the transcriptomes of two non-stimulatory cell lines, HEK293FT and CaCo-2 (https://doi.org/10.6084/m9.figshare.30814700). All of them showed a broad expression in different tissues and cell lines, including keratinocytes, fibroblasts and cell lines, which did not stimulate the Vα3S1/Vβ13S1 TCR (Table S4). This supports that the formation of stimulatory peptides from the parental proteins for presentation is cell type-dependent, probably involving differential processing by the respective cell-type-specific proteasome.

### 3.9. The TiPARP Peptide Stimulated the Vα3S1/Vβ13S1 TCR Both as a Plasmid-Encoded and Exogenously Applied Synthetic Peptide

To further evaluate the antigenicity of the self-peptides, we stimulated the Vα3S1/Vβ13S1 TCR with corresponding synthetic peptides presented by HLA-C*06:02^+^ WM278 cells or the HLA-C*06:02-transfected BCL 721.221, which can present exogenously added synthetic peptide antigens to the Vα3S1/Vβ13S1 TCR [15]. As in previous experiments [13,39], the ADAMTSL5 peptide and an irrelevant peptide isolated from HLA-C*06:02 of an EBV-transformed BCL (FALK peptide [30]) served as positive or negative controls.

When employed as synthetic peptides, the TiPARP peptide stimulated the Vα3S1/Vβ13S1 TCR upon presentation by both WM278 cells (Figure 5C) and HLA-C*06:02^+^ 721.221 cells (Figure 5D). While it activated a lower percentage of Vα3S1/Vβ13S1-TCR hybridoma cells than the autoantigenic ADAMTSL5 peptide, it exhibited a stronger stimulatory capacity as it elicited a higher mean fluorescence intensity (MFI) in the activated hybridoma cells (Figure 5E,F). The difference to the synthetic ADAMTSL5 peptide can be attributed to the high hydrophobicity of the TiPARP peptide, which has an exceptionally strong binding affinity to HLA-C*06:02 as assessed by NetMHCpan-4.1 (Table S7), while the high content of hydrophobic amino acids makes it almost insoluble in aqueous solutions. Therefore, addition of DMSO was required to improve the solubility in aqueous milieu, although this may impair T-cell activation at the same time [46]. The particular stimulatory capacity of the plasmid-encoded peptides, which failed to adequately activate the Vα3S1/Vβ13S1-TCR hybridoma as synthetic peptide, may involve the fact that HLA class I molecules primarily present peptides derived from cytosolic proteins, while exogenously supplied peptides may require cross-presentation and thus are not mandatorily introduced into the HLA class I-presentation pathway [47]. However, differentiating the presentation pathways was not within the scope of this study. Regardless of this, our data show that the Vα3S1/Vβ13S1-TCR can recognise different self-peptides derived from the HLA-C*06:02 immunopeptidome of B cells. Of these, the TiPARP peptide proves to be a genuine B-cell autoantigen of the Vα3S1/Vβ13S1 TCR, which can be presented by HLA-C*06:02 both upon cytosolic expression and exogenous peptide supply.

### 3.10. Binding Prediction Reveals That Other HLA Class I Alleles Associated with Psoriasis May Also Present the Vα3S1/Vβ13S1 TCR Ligands

Other HLA class I alleles associated with a risk of psoriasis are HLA-C*07:01, HLA-C*07:02, HLA-C*07:04, and HLA-B*27:05 [48,49,50,51,52,53], with HLA-B*27:05 and HLA-C*06:02 being phenotype-defining risk alleles also for psoriatic arthritis [48,54]. HLA-C*12:02 and HLA-C*12:03 are susceptibility alleles in Japanese and Turkish ethnical populations [51,55]. Based on their main anchor specificities, HLA-C*06:02, HLA-C*07:01 and HLA-C*07:02 are assigned to the B*27 supertype, presenting overlapping peptide repertoires [38]. We employed NetMHCpan 4.1 (https://services.healthtech.dtu.dk/services/NetMHCpan-4.1/ last accessed on 1 September 2025) to predict the MHC-class I binding of the Vα3S1/Vβ13S1 TCR ligands to these HLA class I molecules. HLA-A*02:01 was used as an unassociated reference allele. As shown in Table S7, most Vα3S1/Vβ13S1 TCR ligands were determined as strong binders to the HLA class I molecules belonging to the B*27 supertype, but usually not HLA-C*12:02, HLA-C*12:03 or HLA-A*02:01. The RNF111 octamer, identified in the non-binding fraction of the immunopeptidome of the HG00131 BCL (Table 1), was only evaluated as an HLA-B*27 binder, although it stimulated the Vα3S1/Vβ13S1 TCR when presented by HLA-C*06:02. False negatives, i.e., peptides that are predicted as non-binders but are experimentally confirmed to stimulate T-cell responses, have been documented [56]. In these instances, experimentally validated immunogenicity is considered essential and decisive for the definitive determination of peptide presentation [57].

## 4. Discussion

The data presented here provide new insights into the pathomechanisms that may transform the main phenotype-specific risk association of psoriasis with HLA-C*06:02 into an active autoimmune disease. They suggest that the pro-inflammatory environment of streptococcal tonsillopharyngitis may enable B cells in the tonsils of HLA-C*06:02^+^ individuals to prime autoreactive CD8^+^ T cells by various self-peptides from the B-cell immunopeptidome, which subsequently cross-react against melanocytes in the skin and trigger psoriasis. This pathogenetic sequence is evident from the dual cellular reactivity of the Vα3S1/Vβ13S1 TCR. This TCR originates from a psoriatic CD8^+^ T-cell clone expanded in lesional epidermis and reacts against HLA-C*06:02^+^ melanocytes by recognizing a peptide derived from ADAMTSL5 as a melanocyte autoantigen [13]. We now find that this TCR is also stimulated by HLA-C*06:02^+^ B cells originating from both tonsils and peripheral blood of psoriasis patients. No other cell type, including dendritic cells, had a stimulatory effect. This is of particular interest, as dendritic cells in particular had been attributed a special stimulatory role in psoriasis [58]. This indicates that B cells may selectively activate the psoriatic autoimmune response of CD8^+^ T cells against melanocytes. Support for this conclusion comes from the HLA class I-restricted autostimulatory property of B cells for autologous CD8^+^ T cells in psoriasis patients, even though we could not demonstrate that this is specifically restricted by HLA-C*06:02 due to a lack of an HLA-C*06:02-specific blocking antibody.

To search for antigens of the Vα3S1/Vβ13S1 TCR presented by HLA-C*06:02 on B cells, we combined TCR reactivity with immunopeptidomics [59]. For this purpose, we isolated the immunopeptidomes presented by HLA-C*06:02 from four different HLA-C*06:02 homozygous BCLs. It was not our intention to perform a comparative analysis of the immunopeptidomes of different cell lines and the effect of different ERAP1 haplotypes on the spectrum of peptides presented by HLA-C*06:02, as this would not have been valid with the methods chosen. Instead, we aimed to isolate as many HLA-C*06:02 peptides from the four BCLs as possible that could be potential antigens of the Vα3S1/Vβ13S1 TCR. Analysis of the four immunopeptidomes illustrated the broad spectrum of self-peptides presented by HLA-C*06:02 [37,38]. The peptide yield of the individual BCLs corresponded to the level of HLA-C*06:02 expression and the degree of Vα3S1/Vβ13S1 TCR stimulation. Using the precisely characterized peptide recognition motif, we were able to identify various self-peptides within the B cell-immunopeptidomes capable of ligating the Vα3S1/Vβ13S1 TCR beyond the ADAMTSL5 peptide. None of the stimulating peptides was present in the immunopeptidomes of HLA-C*06:02-721.221 and C1R cells that did not stimulate Vα3S1/Vβ13S1 TCR, although HLA-C*06:02-721.221 cells were able to present synthetic (Figure 5D–F) or plasmid-encoded ADAMTSL5 peptide upon transfection, suggesting a mutagenesis-altered immunopeptidome in these cell lines.

The identification of the stimulatory peptides in the immunopeptidomes of HLA-C*06:02-homozygous BCLs and the presence of the parental proteins in the BCL transcriptome and in the proteome of B cells, as determined in the Human Protein Atlas, clearly demonstrate that these peptides are B cell-derived autoantigens for the Vα3S1/Vβ13S1 TCR. Accordingly, the reactivity of Vα3S1/Vβ13S1 TCR, which reveals the antigen specificity of a lesionally expanded psoriatic CD8^+^ T-cell clone, suggests that a diverse group of self-peptides derived from the B-cell proteome and presented by HLA-C*06:02 may ultimately trigger the autoimmune response of CD8^+^ T cells against melanocytes in psoriasis. Such a broad immunogenicity of a defined cell type for an individual autoreactive TCR has not yet been observed in autoimmune responses. It highlights the relevance of TCR polyspecificity in autoimmunity, which has been extensively discussed but remains unproven for HLA class I-associated human autoimmune diseases [60,61].

B cells are professional antigen-presenting cells (APCs) with a well-established role in autoimmunity. Besides the production of autoantibodies, they can activate CD4^+^ T cells by processing and presenting self-antigens acquired through antigen-specific B cell-receptors, immune complexes, or pinocytosis via the HLA class II pathway. Such a mechanism is likely involved in the pathogenesis of several autoimmune diseases, such as in multiple sclerosis, where self-peptides presented by B cells via the HLA class II-risk allele HLA-DR15 drive B-T cell interactions in the inflammation of the central nervous system [62,63,64]. In a tumor environment, B cells can enhance CD8^+^ T-cell responses through cross-presentation of tumor antigens [65]. Our results now reveal another, previously unknown pathway, in which B cells might trigger a cross-reactive autoimmune response against another cell type and induce autoimmunity in a distant tissue by stimulating autoreactive CD8^+^ T cells via self-peptides from the B-cell proteome that are presented by a disease-associated HLA class I molecule. The autoantigenic peptides can also be presented by other HLA class I molecules associated with psoriasis (Table S7), suggesting that they may have antigenicity in the pathogenesis of psoriasis beyond HLA-C*06:02.

Sequence comparison revealed no homologies between the B-cell autoantigens ligating the Vα3S1/Vβ13S1 TCR and *S. pyogenes* proteins (Taxid: 1314, analysed with Protein Blast). Further, the Vα3S1/Vβ13S1 TCR was stimulated by HLA-C*06:02^+^ B cells of various origins, and blood-derived B cells from psoriasis patients induced autoproliferation of CD8^+^ T cells in the autologous mixed lymphocyte reaction. These findings make the involvement of streptococcal superantigens or molecular mimicry between *S. pyogenes* antigens and self-peptides as primary drivers of the psoriatic autoimmune response against melanocytes less likely—mechanisms that had previously been proposed as factors for psoriasis onset [20,21].

Instead, the IFN-γ-induced increase in B-cell immunogenicity is compatible with an alternative mechanism for the induction of the psoriatic autoimmune response by streptococcal tonsillopharyngitis. Naive CD8^+^ T cells require substantially higher concentrations of antigenic peptide in conjunction with significantly stronger co-stimulatory signals to become activated compared to antigen-experienced or memory CD8^+^ T cells. Accordingly, naive autoreactive T cells typically remain quiescent unless they encounter sufficient co-stimulatory signals, such as in the context of infections or inflammation [66,67,68]. The pro-inflammatory environment generated during streptococcal tonsillopharyngitis in the tonsils could create co-stimulatory conditions that overcome peripheral tolerance by exceeding the high activation threshold of naive autoreactive CD8^+^ T cells, thereby enabling their activation and clonal expansion by various self-peptides presented by HLA-C*06:02 on tonsillar B cells. Once activated, these autoreactive CD8^+^ T cells may enter the bloodstream and infiltrate the skin, where they cross-react against melanocytes and initiate psoriasis. The role of infection-induced proinflammatory signals would further explain why viral and bacterial infections other than streptococci can also trigger psoriasis [69]. New onset or exacerbation of psoriasis following vaccinations could also be attributable to proinflammatory signals [70,71]. Once the inflammatory stimuli have subsided, a variety of antigens from the environment and an intestinal dysbiosis characterized by altered microbiota composition may promote the persistence of the psoriatic autoimmune response [39,72]. Accordingly, psoriasis patients may benefit from the positive regulatory effects of probiotics and postbiotics on the gut microbiome [73].

This pathogenetic cascade is supported by clinical observations showing that interventions targeting T-cell trafficking can significantly improve psoriasis. Blocking T-cell egress from secondary lymphoid organs by an S_1_P_1_ receptor modulator, preventing the extravasation of T cells into inflamed psoriatic skin by a CD11a/LFA-1 antibody, or eliminating the primary stimulatory site by tonsillectomy, may all lead to disease improvement [17,23,74,75,76]. Accordingly, stimulation of autoreactive T cells by B cells within secondary lymphoid organs may constitute a source of pathogenic CD8^+^ T cells that initiate and maintain psoriatic inflammation. This is reflected in significantly elevated frequencies of ADAMTSL5-specific CD8^+^ T cells circulating in the blood of psoriasis patients [39].

It has been repeatedly argued that the expression of a cellular autoantigen such as ADAMTSL5 must be specific to a particular cell type [77]. Our data suggest that the cell-type-specific processing of the respective protein and not its overall expression determines the immunogenic potential of a specific cell type [78]. While the parental proteins of the self-peptides ligating the Vα3S1/Vβ13S1 TCR are widely expressed (Table S4), only B cells and melanocytes stimulated the Vα3S1/Vβ13S1 TCR. MHC class I-presented peptides originate from proteasomal degradation of cytoplasmic proteins. The differential cell-type-specific expression of proteasome subtypes, which include the standard proteasome, immunoproteasome, and intermediate mixed proteasome, leads to different cell-type and tissue-type-specific immunopeptidomes even when the protein expression profiles overlap [79,80,81,82,83]. B cells constitutively express the immunoproteasome and intermediate proteasome, reflecting their specialized role as professional APCs in immune surveillance and antigen processing [84,85,86]. Consequently, B cells generate a distinct repertoire of immunogenic peptides for presentation than cell types expressing only the standard proteasome [87,88]. A similar mechanism likely governs the generation of the autoantigenic ADAMTSL5 peptide, which selectively activates the Vα3S1/Vβ13S1 TCR in melanocytes but not in other cell types, even when these are conditioned to overexpress the parental ADAMTS-like protein 5 by transfection [13]. Various studies have shown IFN-γ expands the repertoire of peptides available for MHC-class I presentation in melanocytes by upregulating the expression of the immunoproteasome alongside ERAP1, TAP1, and HLA class I molecules, thereby significantly enhancing the immune recognition of melanocytes by T cells and increasing their immunogenic potential [89,90,91]. Correspondingly, the immunogenicity of melanocytes for the Vα3S1/Vβ13S1 TCR was dependent on IFN-γ [13,14]. The increase in the stimulation capacity of native B cells by IFN-γ reflects this effect on the generation and presentation of the peptides that are antigenic for Vα3S1/Vβ13S1 TCR.

Interestingly, B cells from HLA-C*06:02^+^ healthy subjects barely stimulated the Vα3S1/Vβ13S1 TCR and had no consistent autostimulatory effect for CD8^+^ T cells. Stimulation of the Vα3S1/Vβ13S1-TCR hybridoma occurs solely by HLA-C*06:02-presented peptide antigens. Accordingly, B cells of healthy HLA-C*06:02 carriers obviously do not generate and present sufficient amounts of peptides that are antigenic for the Vα3S1/Vβ13S1 TCR. This may also apply to the tonsillar B cells of healthy individuals, although this cannot be tested due to the unavailability of corresponding material, as there is no indication for tonsillectomy in these cases.

Insufficient generation of self-peptides in B cells, which can cross-activate an autoimmune response against melanocytes, due to differences in proteasomal antigen processing and ERAP1 haplotypes, could be one reason that only a fraction of HLA-C*06:02 carriers eventually develop psoriasis [92]. The *ERAP1* gene exhibits various single-nucleotide polymorphisms that combine to form 10 different major haplotypes (Hap1-Hap10) with different enzymatic activities, which influence the generation of peptides in the endoplasmic reticulum for antigen presentation through generation or destruction of peptide ligands [93]. The risk of psoriasis is increased by epistasis between HLA-C*06:02 and the enzymatically highly active ERAP1 haplotype Hap2, while the low-activity haplotype Hap10 protects against psoriasis [29,94]. In previous work, we showed that the generation of the autoantigenic ADAMTSL5 peptide is ERAP1-dependent and that ERAP1 Hap2 increases, while Hap10 reduces both its formation from peptide precursors and the immunogenicity of melanocytes for the Vα3S1/Vβ13S1 TCR [14]. Thus, the extent to which the formation of the B-cell peptides that are antigenic for the Vα3S1/Vβ13S1 TCR is influenced by protective or risk-associated ERAP1 haplotypes, which were expressed in different combinations in the stimulatory BCLs (Table 1), needs to be investigated in further studies.

A limitation of this study is that the main results are based on the analysis of the TCR from a single lesional psoriatic CD8^+^ T-cell clone. However, this is outweighed by several observations. The Vα3S1/Vβ13S1 TCR is pathognomic of the psoriatic autoimmune response in that it rearranges the Vβ13S1-variable region gene in the TCR β-chain (Arden nomenclature [95], corresponding to Vβ 6-5 IMGT gene designation), which is preferentially utilized by the clonal CD8^+^ T cells infiltrating the epidermis of psoriasis lesions [6]. The presence of the Vα3S1/Vβ13S1 TCR in two different psoriasis plaques from the same patient showed that it was specifically selected in the lesional psoriatic infiltrate [7]. Comprehensive immunohistological analyses had revealed that the epidermal CD8^+^ T cells in psoriasis plaques are in direct contact with melanocytes and are activated by them, as demonstrated by the polarisation of cytokine granules at the contact sites [13]. Thus, the reactivity of the Vα3S1/Vβ13S1 TCR reflects the lesional psoriatic T-cell response against melanocytes. Significantly increased frequencies of ADAMTSL5-specific CD8^+^ T cells and high ADAMTSL5 serum autoantibody titers in the blood of psoriasis patients further emphasize the role of ADAMTSL5 as a psoriasis autoantigen [13,39,96,97]. Given that B cells stimulate CD8^+^ T cells in the autologous mixed lymphocyte reaction of psoriasis patients, it may therefore be assumed that the reactivity of the Vα3S1/Vβ13S1 TCR against B cells is representative of the psoriatic autoimmune response.

## 5. Conclusions

Overall, combining the analysis of the HLA class I restriction and the cellular reactivity of a pathogenic TCR with immunopeptidomics enabled the identification of target cells and autoantigens that may activate the lesional psoriatic autoimmune response, as recently outlined [59]. The immunogenicity of the self-peptides that stimulate the Vα3S1/Vβ13S1 TCR for CD8^+^ T cells from psoriasis patients must now be analyzed in stimulation experiments in a follow-up project. Only then can their significance for the pathogenesis of psoriasis be definitively validated. Regardless of this, the HLA-C*06:02-restricted cross-reactivity of the psoriatic Vα3S1/Vβ13S1 TCR against B cells and melanocytes and the auto-stimulatory property of B cells for CD8^+^ T cells from psoriasis patients support the notion that B cells in secondary lymphoid organs might activate CD8^+^ T cells, which subsequently mediate the psoriatic autoimmune response against melanocytes in the skin. CD8^+^ T-cell priming by B cells may require costimulatory signals from a proinflammatory environment, such as the intense inflammation during streptococcal tonsillopharyngitis, which may be enhanced by psoriasis-associated gene variants affecting regulators of innate host defense [98] and further involve B-cell-related psoriasis susceptibility loci [99] and imbalanced B-cell production of pro- and anti-inflammatory cytokines [24,25,26]. The induction of a cross-reactive autoimmune response against another cell type by the HLA class I-presented immunopeptidome of B cells due to TCR polyspecificity reported here reveals a pathogenic pathway, which might also be relevant for other HLA class I-associated inflammatory diseases, such as ankylosing spondylitis, in which changes in B-cell subpopulations and B-cell activation occur [26,100,101,102]. Our findings may further open up potential avenues for the development of therapies that target the pathogenic B-T cell interaction in psoriasis. Eliminating the stimulating conditions for pathogenic T cells by tonsillectomy, which can induce remission in cases of recurrent psoriasis triggered by streptococcal tonsillopharyngitis [17,23,103], supports such a strategy.

## Figures and Tables

**Table 1:** Characteristics of HLA-C*06:02 immunopeptidomes, ERAP1 haplotypes and stimulatory peptides from four HLA-C*06:02-homozygous B-cell lines.

BCL	Total Peptide Yield	HLA-C*06:02 8-mers	HLA-C*06:02 9-mers	HLA-C*06:02 10-mers	HLA-C*06:02 11-mers	ERAP1 Haplotype	Stimulatory Peptides
GM11930	3237	15 (6.15%)	229 (93.85%)	0 (%)	0 (%)	Hap2/Hap2	FGR, CIZ1
HG00142	2144	5 (2.45%)	199 (97.55%)	0 (%)	0 (0%)	Hap10/Hap10	RANBP2, ETF1
GM20771	8648	14 (2.53%)	534 (96.56%)	5 (0.90%)	0 (0%)	Hap2/Hap10	TiPARP, TPGS1
HG00131	8474	16 (4.12%)	370 (95.36%)	1 (0.25%)	1 (0.25%)	Hap1/Hap2	FGR, RNF111

## Data Availability

The mass spectrometry data (immunopeptidomes of *HLA-C*06:02*-positive B-cell lines) can be retrieved via: https://doi.org/10.6084/m9.figshare.30608489. Transcriptome analysis is provided via: https://doi.org/10.6084/m9.figshare.30814700**.** All other data is available in the main text or the Supplementary Materials, or can be requested from the authors.

## Supplementary Materials

The following supporting information can be downloaded at: https://www.mdpi.com/article/10.3390/cells14242002/s1. Table S1. Psoriasis patients and healthy donors; Table S2. HLA class I haplotypes of various EBV-transformed B-cell lines and the corresponding Vα3S1/Vβ13S1-TCR hybridoma activation induced by co-culture with these cell lines; Table S3. Antibodies used in the study; Table S4. Stimulation of the Vα3S1/Vβ13S1 TCR by either naturally *HLA-C*06:02*^+^ or HLA-C*06:02-transfected primary human cells or cell lines and expression patterns of the parental proteins of the antigenic peptides in these cell types; Table S5. Amino acid sequences of 29 self-peptides selected as candidate antigens for the Vα3S1/Vβ13S1 TCR from HLA-C*06:02 immunopeptidomes of HLA-C*06:02-C1R and HLAC*06:02-721.221 and sequences of forward and reverse cDNA oligonucleotides for cloning as minigenes; Table S6. Amino acid sequences of 81 self-peptides selected as candidate antigens for the Vα3S1/Vβ13S1 TCR from the HLA-C*06:02 immunopeptidomes eluted from four homozygous EBV-transformed B-cell lines and sequences of forward and reverse cDNA oligonucleotides for cloning as minigenes; Table S7. Binding affinity of the autoantigenic self-peptides from the B-cell immunopeptidome to various HLA class I molecules as determined by NetMHCPan 4.1; Figure S1. Unprocessed source photographs for the merged photographic images in light microscopy and fluorescence microscopy for Figure 1A and Figure 2A; Figure S2. Antibody markers and gating strategies in FACSorting of PBMCs; Figure S3. Analysis and gating strategy of Vα3S1/Vβ13S1-TCR hybridoma activation by the naturally *HLA-C*06:02*^+^ BCL D22, *HLA-C*06:02*^+^ primary B cells and the irradiation-modified HLA-C*06:02-transfected BCLs 721.221 and C1R; Figure S4. Workflow for assessing cell proliferation of PBMCs in vitro in three sequential steps by different assays; Figure S5. Sequence logos of the Vα3S1/Vβ13S1-TCR peptide recognition motifs and screening motifs and of the HLA-C*06:02 immunopeptidomes; Supplementary Data Sets: HLA_peptides_4_B_cell_lines_FDR_0.1.xlsx; HLA_peptides_4_B_cell_lines_FDR_0.01.xlsx.

## Abbreviations

The following abbreviations are used in this manuscript:

EBV	Epstein–Barr Virus
BCL	B-cell line
AMLR	autologous mixed lymphocyte reaction
AP	autoproliferation
PV	psoriasis patient
HC	healthy control
ERAP1	endoplasmic reticulum aminopeptidase 1
sGFP	superfolder green fluorescent protein
MFI	mean fluorescence intensity
FDR	false discovery rate
