# Supplementary material for: B Cells Can Trigger the T-Cell-Mediated Autoimmune Response Against Melanocytes in Psoriasis
Source: Cells. 2025 Dec 16;14(24):2002. doi: 10.3390/cells14242002 (PMC12732238; doi:10.3390/cells14242002)
Supplement: Supplementary file 1 [file cells-14-02002-s001.zip › Suppl_Tables_Figures_2025_12_04.pdf]

Supplementary Information to the manuscript:

B cells can trigger the T-cell mediated autoimmune response against melanocytes in psoriasis

Authors: Mengwen He, Melissa Bernhardt, Akiko Arakawa, Song-Min Kim, Sigrid Vollmer, Burkard Summer, Yukiyasu Arakawa, Tatsushi Ishimoto, Andreas Schlosser, Jörg Christoph Prinz

Supplementary Tables 1-7

Supplementary Figures 1-5

Supplementary Table S1. Psoriasis patients and healthy donors.

Numbers	Age	Gender	Donated samples
HC01	50	Female	blood
HC02	84	Male	blood
HC03	62	Female	blood
HC04	65	Male	blood
HC05	43	Female	blood
HC06	30	Female	blood
HC07	40	Male	blood
HC08	32	Male	blood
HC09	51	Male	blood
HC10	55	Female	blood
HC11	22	Female	blood
HC12	36	Male	blood
HC13	61	Female	blood
PV01	29	Male	tonsil
PV02	21	Female	tonsil
PV03	33	Female	tonsil
PV04	43	Male	blood
PV05	51	Female	blood
PV06	53	Male	blood
PV07	72	Male	blood
PV08	33	Female	blood
PV09	71	Female	blood
PV10	55	Male	blood
PV11	75	Male	blood
PV12	53	Male	blood
PV13	19	Male	blood
PV14	44	Male	blood
PV15	34	Male	blood
PV16	54	Female	blood
PV17	56	Female	blood
PV18	27	Male	blood
PV19	23	Female	blood
PV20	48	Female	blood
PV21	48	Female	blood
PV22	28	Male	blood
PV23	35	Male	blood
PV24	68	Male	blood

*HC: healthy controls; PV: patients with psoriasis vulgaris

Supplementary Table S2. HLA-class I haplotypes of various EBV-transformed B cell lines and the corresponding Vα3S1/Vβ13S1-TCR hybridoma activation induced by co-culture with these cell lines.

Cell lines	HLA-A		HLA-B		HLA-C		Hybridoma activation
Fey	0101	0101	0801	0801	0701	0701	**–**
P16488	2601	0201	3801	0801	1203	0701	**–**
P17490	0301	0201	3501	4001	03	04	**–**
PSO7	0235	3001	4427	1302	**0602**	0704	**++**
D22	02	02	57	57	**0602**	**0602**	**++**
e9453	0301	0201	35	37	**0602**	0401	**++**
GM20771	0101	0301	1302	5701	**0602**	**0602**	**+++**
GM11930	0201	2402	1302	1302	**0602**	**0602**	**+**
HG00131	0101	0301	5701	3701	**0602**	**0602**	**+++**
HG00142	0201	2902	5701	4501	**0602**	**0602**	**++**
GM12286	0201	2402	1302	5701	**0602**	**0602**	**++**
GM12046	0101	0205	5701	5001	**0602**	**0602**	**+**
HLA-C*06:02-transf. P16488	2601	0201	3801	0801	1203 **0602**	0701	**+**

The degree of TCR hybridoma activation is indicated from moderate to strong by "+" to "+++" (+, up to 30%, ++ 31% up to 60%, +++, >60% of the maximum activation induced by CD3 crosslinking in the same experiment. No activation is denoted by "-", as determined by multiparametric flow cytometry analysis. HLA-C*06:02-P16488 cell line gained stimulatory capacity by transfection with HLA-C*06:02. PSO7 had been established from the donor of the Vα3S1/Vβ13S1 TCR.

Supplementary Table S3. Antibodies used in the study.

A. Unlabeled primary monoclonal antibodies

Specificity	Clone	Supplier	Catalogue number	Final concentration
Purified anti-mouse CD3 antibody	17A2	Biolegend	100202	2 µg/ml
Purified anti-human CD3 antibody	OKT3	Biolegend	317302	2 µg/ml
Ultra-LEAF™ Purified anti-human HLA-A, B, C antibody	W6/32	Biolegend	311428	10 µg/ml
Ultra-LEAF™ Purified Mouse IgG2a	MOPC-173	Biolegend	400263	10 µg/ml

B. Conjugated antibodies used for flow cytometry and FACSorting

Specificity	Label	Clone	Supplier	Catalogue number	Final concentration
anti-human CD20	FITC	2H7	eBioscience	MA1-10136	1:100
anti-human CD4	PE	OKT4	eBioscience	12-0048-42	1:100
anti-human CD8	PerCP/Cyanine 5.5	SK1	Biolegend	344710	1:100
anti-human CD56	PE/Cyanine7	HCD56	Biolegend	318318	1:100
anti-human CD11c	FITC	3.9	Biolegend	301604	1:100
anti-human CD304 (Neuropilin 1)	PE	EPR3113	abcam	ab209445	1:2500
anti-human HLA-DR	PerCP/Cyanine 5.5	L243	Biolegend	307630	1:100
anti-human CD14	APC	63D3	Biolegend	367118	1:100
anti-human CD3	PE	UCHT1	Biolegend	980008	1:100
anti-human CD19	APC	HIB19	Biolegend	982406	1:100
anti-human CD8	PE	SK1	Biolegend	980902	1:100
anti-human CD4	PerCP/Cyanine 5.5	SK3	Biolegend	980810	1:100
anti-human CD45	PerCP/Cyanine 5.5	HI30	Biolegend	304028	1:100
anti-human HLA-C	PE	DT-9	BD Biosciences	566372	1:100
anti-human HLA-DR	Alexa Fluro 647	L243	Biolegend	307622	1:100
mouse IgG2b	PE	MPC-11	BD Biosciences	559529	2.5 µg/ml
mouse IgG2a	PE	MOPC-173	BD Biosciences	565363	2.5 µg/ml

Supplementary Table S4. Stimulation of the Vα3S1/Vβ13S1 TCR by either naturally HLA-C*06:02^{+} or HLA-C*06:02-transfected primary human cells or cell lines and expression patterns of the parental proteins of the antigenic peptides in these cell types.

Cell types/lines	Origin	*HLA-C*06:02* status	ADAMTSL5	TiPARP	FGR	RANBP2	CIZ1	TPGS1	RNF111	ETF1	Vα3S1/Vβ13S1 TCR stimulation
**Primary cells**											
Melanocytes	Primary melanocytes	*HLA-C*06:02*-heterozygous	+	+	-	+	+	+	+	+	+
Keratinocytes	Primary human keratinocytes	*HLA-C*06:02*-transfected	(+)	+	+	+	+	+	+	+	–
Fibroblasts	Primary human skin fibroblasts	*HLA-C*06:02*-heterozygous	+	+	+	+	+	+	+	+	–
B cells	Primary human B cells	*HLA-C*06:02*-heterozygous	-	+	+	+	+	+	+	+	+
**Cell lines**											
PSO7	Autologous EBV-transformed B-LCL, positive control	*HLA-C*06:02*-heterozygous	(+)	+	+	+	+	(+)	+	+	+
HaCaT	Spontaneously immortalized human keratinocyte cell line	HLA-C*06:02-transfected	+	+	(+)	+	+	+	+	+	–
A-431	Human epidermoid carcinoma	HLA-C*06:02-transfected	+	+	-	+	+	+	+	+	–
HUVEC	Primary umbilical vein endothelial cells	HLA-C*06:02-transfected	+	+	-	+	+	(+)	+	+	–
NCI-H1975	Human non-small cell lung cancer cell	HLA-C*06:02-transfected	+	+	-	+	+	-	-	+	–
CaCo2	Human epithelial colorectal adenocarcinoma	HLA-C*06:02-transfected	+	+	-	+	+	(+)	+	+	–
MCF-7	Human breast carcinoma cell	HLA-C*06:02-transfected	+	+	-	+	+	+	+	+	–
KHOS	Osteosarcoma	HLA-C*06:02-transfected	+	+	-	+	+	-	+	+	–
K-562	Human immortalized myelogenous leukemia cell line	HLA-C*06:02-transfected	+	+	-	+	+	-	+	+	–
U-937	Pro-monocytic, human myeloid Leukemia cell line	HLA-C*06:02-transfected	+	+	(+)	+	+	+	+	+	–
HEK293FT	Human Embryonic Kidney cell line	HLA-C*06:02-transfected	(+)	+	-	+	+	+	+	+	–

Expression of transcripts in the different cell types according to the Human Protein Atlas or transcriptome analysis (B-cell lines, CaCo2 and HEK293FT cell lines): +, yes; (+), weak; -, not detected. TCR hybridoma activation is determined by sGFP induction analyzed by multiparametric flow cytometry analysis and indicated as +, yes ;–, no.

Supplementary Table S5. Amino acid sequences of 29 self-peptides selected as candidate antigens for the Vα3S1/Vβ13S1 TCR from HLA-C*06:02 immunopeptidomes of HLA-C*06:02-C1R and HLA-C*06:02-721.221 and sequences of forward and reverse cDNA oligonucleotides for cloning as minigenes.

Parent protein	B-cell self-peptide	Nucleotide sequences of cDNA inserts
AURKA	LRHPNILRL_for	5'- CACC ATG CTT CGG CAT CCT AAT ATT CTT AGA CTG TGA -3'
	rev	5'- TCA CAG TCT AAG AAT ATT AGG ATG CCG AAG -3'
ALG3	QRAWQERRL_for	5'- CACC ATG CAG CGC GCC TGG CAA GAG CGG CGC CTG TGA -3'
	rev	5'- TCA CAG GCG CCG CTC TTG CCA GGC GCG CTG -3'
AQR	LRNFNLFRL_for	5'- CACC ATG CTA AGG AAC TTT AAC CTC TTC CGC TTA TGA -3'
	rev	5'- TCA TAA GCG GAA GAG GTT AAA GTT CCT TAG -3'
NLRC5	FRPEHVSRL_for	5'- CACC ATG TTC CGG CCA GAG CAC GTG TCC AGG CTG TGA -3'
	rev	5'- TCA CAG CCT GGA CAC GTG CTC TGG CCG GAA -3'
UNC119B	IRPEHVLRL_for	5'- CACC ATG ATC CGG CCC GAG CAC GTC CTG CGC CTC TGA -3'
	rev	5'- TCA GAG GCG CAG GAC GTG CTC GGG CCG GAT -3'
SND1	ERFAKERRL_for	5'- CACC ATG GAG AGG TTT GCC AAA GAG CGC AGG CTG TGA -3'
	rev	5'- TCA CAG CCT GCG CTC TTT GGC AAA CCT CTC -3'
SF3B3	SRPVKLFRV_for	5'- CACC ATG TCC CGT CCT GTG AAG CTC TTC CGA GTC TGA -3'
	rev	5'- TCA GAC TCG GAA GAG CTT CAC AGG ACG GGA -3'
CSNK2A1	LRYDHQSRL_for	5'- CACC ATG CTG CGA TAT GAC CAC CAG TCA CGG CTT TGA -3'
	rev	5'- TCA AAG CCG TGA CTG GTG GTC ATA TCG CAG -3'
NXF1	ERFPKLLRL_for	5'- CACC ATG GAA CGA TTT CCC AAG TTA CTA CGC CTG TGA -3'
	rev	5'- TCA CAG GCG TAG TAA CTT GGG AAA TCG TTC -3'
PSMD2	SRFPEALRL_for	5'- CACC ATG AGC CGC TTC CCT GAA GCT CTG AGA TTG TGA -3'
	rev	5'- TCA CAA TCT CAG AGC TTC AGG GAA GCG GCT -3'
SMG1	KRASYILRL_for	5'- CACC ATG AAA CGT GCA AGT TAC ATC TTG CGT CTT TGA -3'
	rev	5'- TCA AAG ACG CAA GAT GTA ACT TGC ACG TTT -3'
AIFM2	YRKAFESRL_for	5'- CACC ATG TAC CGC AAA GCG TTT GAG AGC AGA CTA TGA -3'
	rev	5'- TCA TAG TCT GCT CTC AAA CGC TTT GCG GTA -3'
PSMD2	SRPELIFRL_for	5'- CACC ATG TCA AGG CCT GAA CTT ATT TTT AGA CTA TGA -3'
	rev	5'- TCA TAG TCT AAA AAT AAG TTC AGG CCT TGA -3'
ZNF282	NRNFWVLRL_for	5'- CACC ATG AAC AGG AAC TTC TGG GTC CTG CGG CTG TGA -3'
	rev	5'- TCA CAG CCG CAG GAC CCA GAA GTT CCT GTT -3'
CD70	SRSISLLRL_for	5'- CACC ATG TCC CGT AGC ATC AGC CTG CTG CGT CTC TGA -3'
	rev	5'- TCA GAG ACG CAG CAG GCT GAT GCT ACG GGA -3'
ACACA	FRNERAIRF_for	5'- CACC ATG TTT CGA AAT GAA CGT GCA ATT AGA TTC TGA -3'
	rev	5'- TCA GAA TCT AAT TGC ACG TTC ATT TCG AAA -3'
ZNF574	VRFHRPYRL_for	5'- CACC ATG GTG CGT TTT CAC CGT CCT TAC CGC CTG TGA -3'
	rev	5'- TCA CAG GCG GTA AGG ACG GTG AAA ACG CAC -3'
VPS51	GAYGQAVRY_for	5'- CACC ATG GGC GCC TAT GGG CAG GCG GTG CGC TAC TGA -3'
	rev	5'- TCA GTA GCG CAC CGC CTG CCC ATA GGC GCC -3'
MFSD5	YRIATSKRY_for	5'- CACC ATG TAC CGT ATC GCC ACC TCC AAG AGG TAC TGA -3'
	rev	5'- TCA GTA CCT CTT GGA GGT GGC GAT ACG GTA -3'
CSNK2A2	LRYDHQQRL_for	5'- CACC ATG CTG CGA TAC GAC CAT CAA CAG AGA CTG TGA -3'
	rev	5'- TCA CAG TCT CTG TTG ATG GTC GTA TCG CAG -3'
PARP14	FRIEKIERI _for	5'- CACC ATG TTC AGA ATA GAG AAG ATT GAG AGG ATC TGA -3'
	rev	5'- TCA GAT CCT CTC AAT CTT CTC TAT TCT GAA -3'
SF284	KLYGKPIRV_for	5'- CACC ATG AAA CTC TAT GGG AAG CCA ATA CGG GTG TGA -3'
	rev	5'- TCA CAC CCG TAT TGG CTT CCC ATA GAG TTT -3'
FBXW8	HRFEHDARI_for	5'- CACC ATG CAT CGT TTT GAG CAC GAT GCA AGA ATA TGA -3'

	rev	5'- TCA TAT TCT TGC ATC GTG CTC AAA ACG ATG -3'
SLC4A1AP	VHVGHVVRF_for	5'- CACC ATG GTC CAC GTT GGG CAT GTT GTT CGC TTT TGA -3'
	rev	5'- TCA AAA GCG AAC AAC ATG CCC AAC GTG GAC -3'
CLASP2	IRHTHVPRL_for	5'- CACC ATG ATT CGG CAT ACT CAT GTA CCC AGA CTT TGA -3'
	rev	5'- TCA AAG TCT GGG TAC ATG AGT ATG CCG AAT -3'
NEMF	VRKDHENRL_for	5'- CACC ATG GTT CGA AAG GAT CAC GAA AAC AGA TTG TGA -3'
	rev	5'- TCA CAA TCT GTT TTC GTG ATC CTT TCG AAC -3'
RP9	KRHEKDVRI_for	5'- CACC ATG AAA CGA CAT GAA AAG GAC GTA AGG ATA TGA -3'
	rev	5'- TCA TAT CCT TAC GTC CTT TTC ATG TCG TTT -3'
MYH4	SRFGKFIRI_for	5'- CACC ATG TCT CGC TTT GGT AAA TTC ATC AGG ATC TGA -3'
	rev	5'- TCA GAT CCT GAT GAA TTT ACC AAA GCG AGA -3'
LSM7	KYIDKTIRV_for	5'- CACC ATG AAG TAC ATC GAC AAG ACG ATC CGG GTA TGA -3'
	rev	5'- TCA TAC CCG GAT CGT CTT GTC GAT GTA CTT -3'

The abbreviation “for” indicates “forward” strand, while “rev” indicates “reverse” strand for cloning of minigenes.

Supplementary Table S6. Amino acid sequences of 81 self-peptides selected as candidate antigens for the Vα3S1/Vβ13S1 TCR from the HLA-C*06:02 immunopeptidomes eluted from four *HLA-C*06:02* homozygous EBV-transformed B cell lines and sequences of forward and reverse cDNA oligonucleotides for cloning as minigenes.

Parent protein	B-cell self-peptide	Nucleotide sequences of cDNA inserts
L3MBTL4	KRNPRLIRV_for	5’- CACC ATG AAA CGG AAC CCC AGG TTA ATT CGT GTT TGA -3’
	rev	5’- TCA AAC ACG AAT TAA CCT GGG GTT CCG TTT -3’
FNDC3B	RQIDRQNRL_for	5’- CACC ATG CGC CAG ATC GAT CGC CAG AAC CGC CTC TGA -3’
	rev	5’- TCA GAG GCG GTT CTG GCG ATC GAT CTG GCG -3’
MRPL20	RVTDRYFRI_for	5’- CACC ATG CGC GTC ACC GAC CGC TAC TTT CGG ATC TGA -3’
	rev	5’- TCA GAT CCG AAA GTA GCG GTC GGT GAC GCG -3’
SNX8	KLRDRAERI_for	5’- CACC ATG AAG CTT CGC GAC AGG GCC GAG CGG ATC TGA -3’
	rev	5’- TCA GAT CCG CTC GGC CCT GTC GCG AAG CTT -3’
CLSTN3	KLYDRILRV_for	5’- CACC ATG AAG CTG TAC GAT CGC ATC CTG CGG GTG TGA -3’
	rev	5’- TCA CAC CCG CAG GAT GCG ATC GTA CAG CTT -3’
TFRC	KLNDRVMRV_for	5’- CACC ATG AAA CTC AAT GAT CGT GTC ATG AGA GTG TGA -3’
	rev	5’- TCA CAC TCT CAT GAC ACG ATC ATT GAG TTT -3’
TOMM34	ALYGRALRV_for	5’- CACC ATG GCG CTC TAC GGC CGC GCG CTG CGG GTG TGA -3’
	rev	5’- TCA CAC CCG CAG CGC GCG GCC GTA GAG CGC -3’
ROCK2	FLTDREVRL_for	5’- CACC ATG TTC TTA ACA GAT AGG GAG GTA CGA CTT TGA -3’
	rev	5’- TCA AAG TCG TAC CTC CCT ATC TGT TAA GAA -3’
LACTB2	RLSNRVVRV_for	5’- CACC ATG CGG CTG TCC AAT CGA GTC GTG CGT GTG TGA -3’
	rev	5’- TCA CAC ACG CAC GAC TCG ATT GGA CAG CCG -3’
RGS9	RYQGRQYRL_for	5’- CACC ATG AGA TAC CAG GGC AGA CAG TAC AGA CTG TGA -3’
	rev	5’- TCA CAG TCT GTA CTG TCT GCC CTG GTA TCT -3’
MOB3A	KILSRLFRV_for	5’- CACC ATG AAG ATC CTG TCG CGG CTG TTC CGC GTG TGA -3’
	rev	5’- TCA CAC GCG GAA CAG CCG CGA CAG GAT CTT -3’
RAPGEF3	ERRRCHRL_for	5’- CACC ATG GAG AGG CGG CGA TGC CAC AGG TTG TGA -3’
	rev	5’- TCA CAA CCT GTG GCA TCG CCG CCT CTC -3’
ARAP1	RVPRAVRV_for	5’- CACC ATG CGA GTC CCA CGG GCC GTG CGC GTG TGA -3’
	rev	5’- TCA CAC GCG CAC GGC CCG TGG GAC TCG -3’
ARRDC4	VALRALRL_for	5’- CACC ATG GTG GCC CTG CGC GCG CTG CGC CTG TGA -3’
	rev	5’- TCA CAG GCG CAG CGC GCG CAG GGC CAC -3’
RPL35	KQQRKERL_for	5’- CACC ATG AAG CAG CAG CGG AAG GAG CGG CTG TGA -3’
	rev	5’- TCA CAG CCG CTC CTT CCG CTG CTG CTT -3’
PHRF1	RISTARRV_for	5’- CACC ATG CGG ATC TCC ACG GCC AGG AGG GTC TGA -3’
	rev	5’- TCA GAC CCT CCT GGC CGT GGA GAT CCG -3’
CELSR1	GVIRTQRRL_for	5’- CACC ATG GGT GTG ATT CGC ACC CAG CGC CGG CTG TGA -3’
	rev	5’- TCA CAG CCG GCG CTG GGT GCG AAT CAC ACC -3’
TOR1AIP1	RSVRAIRI_for	5’- CACC ATG CGT AGT GTC CGT GCC ATC CGG ATT TGA -3’
	rev	5’- TCA AAT CCG GAT GGC ACG GAC ACT ACG -3’
LPCAT1	LYASNVRRV_for	5’- CACC ATG CTG TAT GCC AGC AAC GTG CGG CGA GTC TGA -3’
	rev	5’- TCA GAC TCG CCG CAC GTT GCT GGC ATA CAG -3’
IQSEC1	QYYPNGIRL_for	5’- CACC ATG CAG TAC TAC CCC AAT GGC ATC CGG CTC TGA -3’
	rev	5’- TCA GAG CCG GAT GCC ATT GGG GTA GTA CTG -3’
RASGRP1	KISLRLKRA_for	5’- CACC ATG AAG ATT TCT CTT CGG CTG AAG AGG GCT TGA -3’
	rev	5’- TCA AGC CCT CTT CAG CCG AAG AGA AAT CTT -3’
COMTD1	RIRRDVRV_for	5’- CACC ATG CGC ATC CGG CGG GAC GTC AGG GTC TGA -3’
	rev	5’- TCA GAC CCT GAC GTC CCG CCG GAT GCG -3’
CFAP20	VRNGHIKRI_for	5’- CACC ATG GTA CGG AAT GGC CAC ATC AAA AGA ATC TGA -3’
	rev	5’- TCA GAT TCT TTT GAT GTG GCC ATT CCG TAC -3’
RPS13	ARYYKTKRV_for	5’- CACC ATG GCT CGA TAT TAT AAG ACC AAG CGA GTC TGA -3’
	rev	5’- TCA GAC TCG CTT GGT CTT ATA ATA TCG AGC -3’
CCT2	KMKEKVERI_for	5’- CACC ATG AAA ATG AAG GAG AAA GTT GAA CGT ATT TGA -3’
	rev	5’- TCA AAT ACG TTC AAC TTT CTC CTT CAT TTT -3’
ANXA6	RSYPHLRRV_for	5’- CACC ATG CGG AGC TAT CCG CAC CTC CGG AGA GTC TGA -3’
	rev	5’- TCA GAC TCT CCG GAG GTG CGG ATA GCT CCG -3’
PNISR	RSYSRRIKI_for	5’- CACC ATG AGA AGC TAT TCT CGC AGA ATT AAA ATA TGA -3’
	rev	5’- TCA TAT TTT AAT TCT GCG AGA ATA GCT TCT -3’
DDX18	FSATQTRKV_for	5’- CACC ATG TTT TCT GCC ACC CAA ACT CGA AAA GTT TGA -3’

		rev	5'- TCA AAC TTT TCG AGT TTG GGT GGC AGA AAA -3'
CDC73	RVVDQPLKL	_for	5'- CACC ATG AGA GTA GTA GAC CAG CCC CTT AAA CTT TGA -3'
		rev	5'- TCA AAG TTT AAG GGG CTG GTC TAC TAC TCT -3'
CD180	IRGNVKKL	_for	5'- CACC ATG ATC AGA GGC AAC GTG AAG AAA CTT TGA -3'
		rev	5'- TCA AAG TTT CTT CAC GTT GCC TCT GAT -3'
AC011477.4	KAYKQSSHL	_for	5'- CACC ATG AAA GCC TAT AAG CAG TCC TCA CAC CTT TGA -3'
		rev	5'- TCA AAG GTG TGA GGA CTG CTT ATA GGC TTT -3'
RANBP2	MRREQVLKV	_for	5'- CACC ATG ATG CGA AGA GAA CAA GTA CTA AAA GTG TGA -3'
		rev	5'- TCA CAC TTT TAG TAC TTG TTC TCT TCG CAT -3'
RFC1	KRKENAIKL	_for	5'- CACC ATG AAA AGA AAA GAA AAT GCC ATT AAA TTG TGA -3'
		rev	5'- TCA CAA TTT AAT GGC ATT TTC TTT TCT TTT -3'
POLR3B	SQFEKTRKV	_for	5'- CACC ATG TCC CAG TTT GAA AAA ACG AGA AAA GTG TGA -3'
		rev	5'- TCA CAC TTT TCT CGT TTT TTC AAA CTG GGA -3'
ADAR	MMPNKVRKI	_for	5'- CACC ATG ATG ATG CCC AAC AAG GTC AGG AAG ATT TGA -3'
		rev	5'- TCA AAT CTT CCT GAC CTT GTT GGG CAT CAT -3'
FGR	TRGDHVKHY	_for	5'- CACC ATG ACC AGA GGC GAT CAT GTG AAG CAT TAC TGA -3'
		rev	5'- TCA GTA ATG CTT CAC ATG ATC GCC TCT GGT -3'
CORO1A	VRSSKFRHV	_for	5'- CACC ATG GTC CGC TCC AGC AAG TTC CGC CAC GTG TGA -3'
		rev	5'- TCA CAC GTG GCG GAA CTT GCT GGA GCG GAC -3'
SGO2	KRISKTTKL	_for	5'- CACC ATG AAA AGA ATT TCA AAG ACT ACT AAG TTG TGA -3'
		rev	5'- TCA CAA CTT AGT AGT CTT TGA AAT TCT TTT -3'
CCAR2	FQTSHTLHL	_for	5'- CACC ATG TTC CAA ACA TCC CAC ACA CTT CAC CTG TGA -3'
		rev	5'- TCA CAG GTG AAG TGT GTG GGA TGT TTG GAA -3'
TNFAIP3	HAPDHTRHL	_for	5'- CACC ATG CAC GCC CCA GAC CAC ACA AGG CAC TTG TGA -3'
		rev	5'- TCA CAA GTG CCT TGT GTG GTC TGG GGC GTG -3'
EIF4G1	IIFETPLRV	_for	5'- CACC ATG ATT ATT TTT GAG ACT CCC CTC CGA GTG TGA -3'
		rev	5'- TCA CAC TCG GAG GGG AGT CTC AAA AAT AAT -3'
RRAS	KLFTQILRV	_for	5'- CACC ATG AAG CTC TTC ACG CAG ATT CTG CGG GTC TGA -3'
		rev	5'- TCA GAC CCG CAG AAT CTG CGT GAA GAG CTT -3'
TBC1D30	AAAETELRV	_for	5'- CACC ATG GCT GCA GCT GAA ACT GAG CTC AGG GTG TGA -3'
		rev	5'- TCA CAC CCT GAG CTC AGT TTC AGC TGC AGC -3'
MAU2	AQFTTALRL	_for	5'- CACC ATG GCC CAG TTC ACC ACG GCC CTG CGG CTC TGA -3'
		rev	5'- TCA GAG CCG CAG GGC CGT GGT GAA CTG GGC -3'
PREX1	QYVTQINRL	_for	5'- CACC ATG CAA TAT GTC ACC CAG ATC AAC AGG CTG TGA -3'
		rev	5'- TCA CAG CCT GTT GAT CTG GGT GAC ATA TTG -3'
ZFC3H1	KLQEQIHRV	_for	5'- CACC ATG AAG CTT CAG GAA CAA ATT CAC AGA GTT TGA -3'
		rev	5'- TCA AAC TCT GTG AAT TTG TTC CTG AAG CTT -3'
GINS1	YLYDRLLRI	_for	5'- CACC ATG TAC CTG TAT GAC CGC TTG CTT CGG ATC TGA -3'
		rev	5'- TCA GAT CCG AAG CAA GCG GTC ATA CAG GTA -3'
INTS3	VRFGQQKRY	_for	5'- CACC ATG GTG CGA TTT GGT CAA CAA AAG CGA TAC TGA -3'
		rev	5'- TCA GTA TCG CTT TTG TTG ACC AAA TCG CAC -3'
TIPARP	YRILQILRV	_for	5'- CACC ATG TAC AGA ATT TTG CAG ATA TTG AGA GTC TGA -3'
		rev	5'- TCA GAC TCT CAA TAT CTG CAA AAT TCT GTA -3'
DDX51	YTDATPLRV	_for	5'- CACC ATG TAC ACA GAT GCC ACA CCT CTG AGA GTC TGA -3'
		rev	5'- TCA GAC TCT CAG AGG TGT GGC ATC TGT GTA -3'
KIF4B	RYADRARKI	_for	5'- CACC ATG CGC TAT GCT GAC AGA GCA AGA AAA ATC TGA -3'
		rev	5'- TCA GAT TTT TCT TGC TCT GTC AGC ATA GCG -3'
RBM15	VLYDRPLKI	_for	5'- CACC ATG GTG CTC TAT GAC CGG CCT CTG AAG ATA TGA -3'
		rev	5'- TCA TAT CTT CAG AGG CCG GTC ATA GAG CAC -3'
DIP2B	KLWSRSLKL	_for	5'- CACC ATG AAG TTG TGG AGC AGA AGT TTA AAG TTG TGA -3'
		rev	5'- TCA CAA CTT TAA ACT TCT GCT CCA CAA CTT -3'
HNRNPM	SLSGRPLKV	_for	5'- CACC ATG AGT CTG AGC GGA AGA CCA CTG AAA GTC TGA -3'
		rev	5'- TCA GAC TTT CAG TGG TCT TCC GCT CAG ACT -3'
FYTTD1	FLFRRGLKV	_for	5'- CACC ATG TTT CTT TTC AGA AGA GGC CTG AAG GTG TGA -3'
		rev	5'- TCA CAC CTT CAG GCC TCT TCT GAA AAG AAA -3'
SRP68	VLYDRVLKY	_for	5'- CACC ATG GTC CTG TAT GAC AGA GTC CTG AAA TAT TGA -3'
		rev	5'- TCA ATA TTT CAG GAC TCT GTC ATA CAG GAC -3'
PSMD2	RTFGHLLRY	_for	5'- CACC ATG CGA ACC TTT GGC CAC TTG CTG AGA TAT TGA -3'
		rev	5'- TCA ATA TCT CAG CAA GTG GCC AAA GGT TCG -3'
DNAH17	VLYLKPLRI	_for	5'- CACC ATG GTG CTC TAT TTG AAG CCC CTA CGG ATC TGA -3'
		rev	5'- TCA GAT CCG TAG GGG CTT CAA ATA GAG CAC -3'
DDX6	KLPPKDLRI	_for	5'- CACC ATG AAA CTC CCT CCA AAG GAT CTA AGA ATC TGA -3'

	rev	5’- TCA GAT TCT TAG ATC CTT TGG AGG GAG TTT -3’
ACLY	TFMDHVLRY_for	5’- CACC ATG ACA TTC ATG GAT CAT GTG TTA CGC TAT TGA -3’
	rev	5’- TCA ATA GCG TAA CAC ATG ATC CAT GAA TGT -3’
DST	TYAEKLHRL_for	5’- CACC ATG ACT TAT GCA GAA AAG TTG CAC AGA TTA TGA -3’
	rev	5’- TCA TAA TCT GTG CAA CTT TTC TGC ATA AGT -3’
SCNN1D	REIRLQRL_for	5’- CACC ATG CGG GAG ATC CGT CTG CAG AGG CTG TGA -3’
	rev	5’- TCA CAG CCT CTG CAG ACG GAT CTC CCG -3’
TRIM68	RSQRPVRW_for	5’- CACC ATG AGG TCG CAG AGG CCT GTC CGC TGG TGA -3’
	rev	5’- TCA CCA GCG GAC AGG CCT CTG CGA CCT -3’
KIF20A	RKRQTLRL_for	5’- CACC ATG CGC AAG AGG CAG ACT TTG CGG CTA TGA -3’
	rev	5’- TCA TAG CCG CAA AGT CTG CCT CTT GCG -3’
HELZ2	RTVTPLRW_for	5’- CACC ATG CGG ACG GTC ACG CCT CTG CGG TGG TGA -3’
	rev	5’- TCA CCA CCG CAG AGG CGT GAC CGT CCG -3’
DNAJC30	RSMKGLRW_for	5’- CACC ATG CGG TCC ATG AAA GGC CTC CGC TGG TGA -3’
	rev	5’- TCA CCA GCG GAG GCC TTT CAT GGA CCG -3’
MGRN1	IRKDSLRLV_for	5’- CACC ATG ATC CGC AAA GAC TCC CTG CGG CTG GTG TGA -3’
	rev	5’- TCA CAC CAG CCG CAG GGA GTC TTT GCG GAT -3’
MX2	TRCPLVLKL_for	5’- CACC ATG ACC AGG TGT CCG CTG GTG CTG AAA CTG TGA -3’
	rev	5’- TCA CAG TTT CAG CAC CAG CGG ACA CCT GGT -3’
RNF213	HRVYLVRKL_for	5’- CACC ATG CAC CGG GTG TAC CTG GTG CGG AAG CTC TGA -3’
	rev	5’- TCA GAG CTT CCG CAC CAG GTA CAC CCG GTG -3’
TPGS1	TYSELLRRI_for	5’- CACC ATG ACC TAC AGC GAG CTG CTC AGG CGC ATC TGA -3’
	rev	5’- TCA GAT GCG CCT GAG CAG CTC GCT GTA GGT -3’
CIZ1	RYFKTPRKF_for	5’- CACC ATG CGC TAC TTC AAA ACC CCT CGC AAG TTT TGA -3’
	rev	5’- TCA AAA CTT GCG AGG GGT TTT GAA GTA GCG -3’
EMC2	KYFAQALKL_for	5’- CACC ATG AAG TAT TTT GCA CAG GCA TTG AAA CTG TGA -3’
	rev	5’- TCA CAG TTT CAA TGC CTG TGC AAA ATA CTT -3’
TFRC	REFKLSKV_for	5’- CACC ATG CGT GAA TTT AAA CTC AGC AAA GTC TGA -3’
	rev	5’- TCA GAC TTT GCT GAG TTT AAA TTC ACG -3’
WDR81	RDLRKSKF_for	5’- CACC ATG CGA GAC CTG CGC AAG TCC AAG TTC TGA -3’
	rev	5’- TCA GAA CTT GGA CTT GCG CAG GTC TCG -3’
ETF1	KRHNYVRKV_for	5’- CACC ATG AAG CGA CAT AAC TAT GTT CGG AAA GTA TGA -3’
	rev	5’- TCA TAC TTT CCG AAC ATA GTT ATG TCG CTT -3’
VPS39	KRSQLVKKL_for	5’- CACC ATG AAA CGA AGT CAA TTG GTA AAG AAG CTG TGA -3’
	rev	5’- TCA CAG CTT CTT TAC CAA TTG ACT TCG TTT -3’
STAT6	YQRDPLKL_for	5’- CACC ATG TAT CAG AGG GAC CCC CTG AAG CTG TGA -3’
	rev	5’- TCA CAG CTT CAG GGG GTC CCT CTG ATA -3’
NUP85	VYSQILRKL_for	5’- CACC ATG GTT TAC TCT CAA ATC TTG AGA AAA CTC TGA -3’
	rev	5’- TCA GAG TTT TCT CAA GAT TTG AGA GTA AAC -3’
AL451007.3	RRYLRRKE_for	5’- CACC ATG CGG CGC TAC CTG CGG CGC AAG GAG TGA -3’
	rev	5’- TCA CTC CTT GCG CCG CAG GTA GCG CCG -3’
RNF111	RRLPCRKR_for	5’- CACC ATG CGG AGA CTT CCA TGC AGA AAG AGA TGA -3’
	rev	5’- TCA TCT CTT TCT GCA TGG AAG TCT CCG -3’
CCL22	VRYRLPLRV_for	5’- CACC ATG GTC CGT TAC CGT CTG CCC CTG CGC GTG TGA -3’
	rev	5’- TCA CAC GCG CAG GGG CAG ACG GTA ACG GAC -3’

The abbreviation “for” indicates “forward” strand, while “rev” indicates “reverse” strand for cloning of minigenes. Peptides that stimulate the Vα3S1/Vβ13S1 TCR are highlighted in grey.

Supplementary Table S7. Binding affinity of the autoantigenic self-peptides from the B cell-immunopeptidome to various HLA-class I-molecules as determined by NetMHCPan 4.1.

**Peptid**	**HLA-C*06:02**	**HLA-C*07:01**	**HLA-C*07:02**	**HLA-C*07:04**	**HLA-C*12:02**	**HLA-C*12:03**	**HLA-B*27:05**	**HLA-A*02:01**
**ADAMTSL5**	0.287 <= SB	0.342 <= SB	0.949 <= WB	0.968 <= WB	15.702	8.656	1.064 <= WB	38.800
**TIPARP**	0.007 <= SB	0.073 <= SB	0.187 <= SB	0.185 <= SB	2.963	2.314	0.111 <= SB	6.129
**FGR**	0.034 <= SB	0.021 <= SB	0.035 <= SB	1.459 <= WB	1.401 <= WB	2.132	0.447 <= SB	29.720
**RANBP2**	0.032 <= SB	0.115 <= SB	0.471 <= SB	0.307 <= SB	3.938	3.057	0.386 <= SB	11.641
**CIZ1**	0.070 <= SB	0.065 <= SB	0.011 <= SB	0.200 <= SB	1.201 <= WB	0.867 <= WB	1.082 <= WB	17.897
**TPGS1**	0.111 <= SB	0.604 <= WB	0.500 <= SB	0.296 <= SB	6.373	3.350	9.153	10.742
**RNF111**	19.250	15.526	21.947	52.500	59.000	52.500	1.614 <= WB	71.250
**ETF1**	0.018 <= SB	0.075 <= SB	0.416 <= SB	0.362 <= SB	7.214	1.788 <= WB	0.296 <= SB	12.832

Rank Threshold for Strong binding peptides (SB) 0.500; # Rank Threshold for Weak binding peptides (WB) 2.000. NetMHCpan 4.1 - DTU Health Tech - Bioinformatic Services

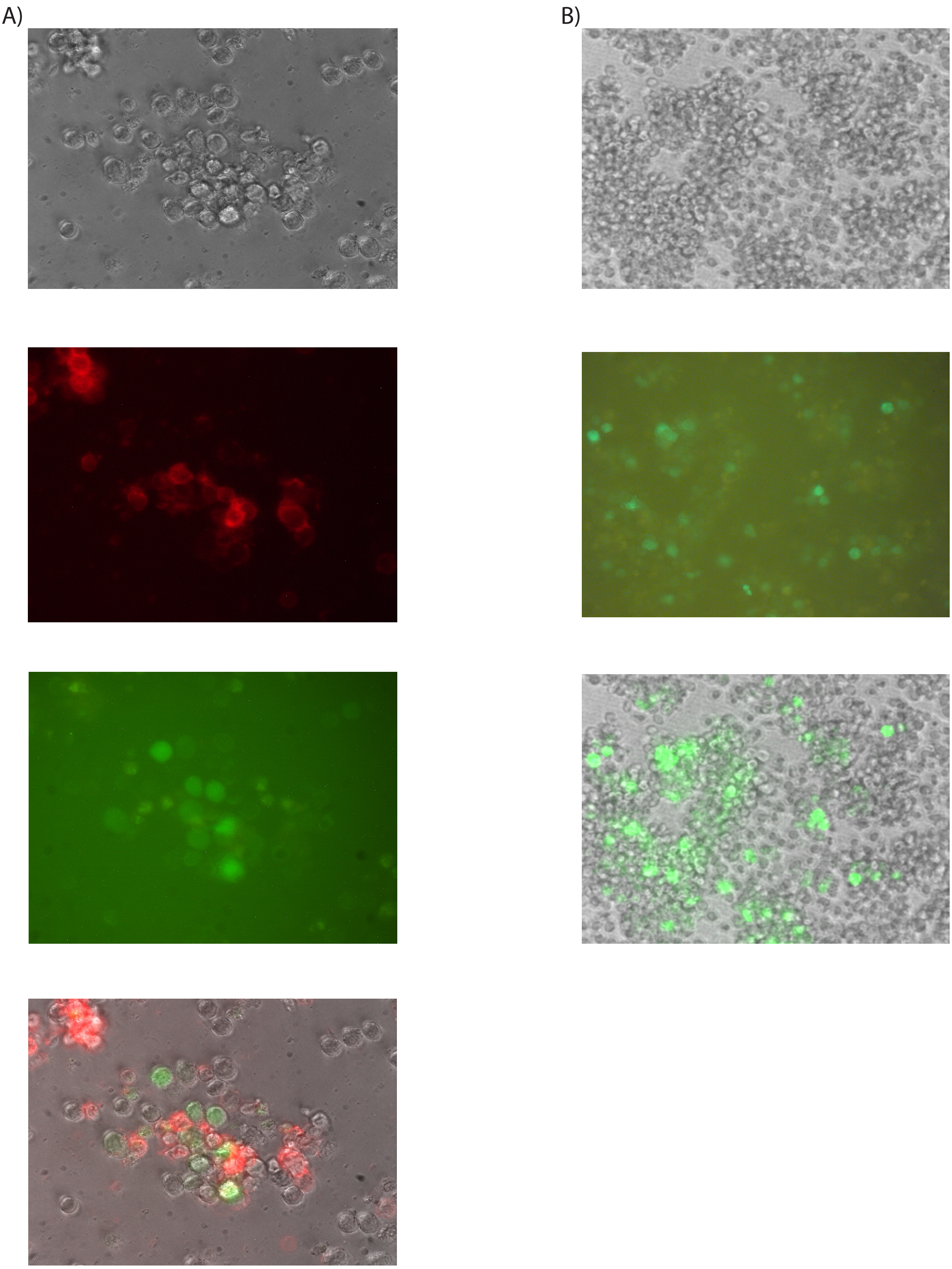

Supplementary Figure S1 | Unprocessed source photographs for the merged photographic images in light microscopy and fluorescen-ce microscopy for Figure 1a and Figure 2a. A) Co-culture of the Vα3S1/Vβ13S1-TCR hybridoma with tonsil cells in light microscopy, induced to express GFP (green) with tonsil cells stained with Alexa Fluor 647-labeled HLA-DR antibody (red), as shown in the merged UV-/light microscopy image
(from top to bottom). **B)** Co-culture of the Vα3S1/Vβ13S1-TCR hybridoma with a *HLA-C*06:02*$^{+}$ B cell line in light microscopy and induced to express GFP (green), as shown in a merged UV-/light microscopy image (from top to bottom).

A)

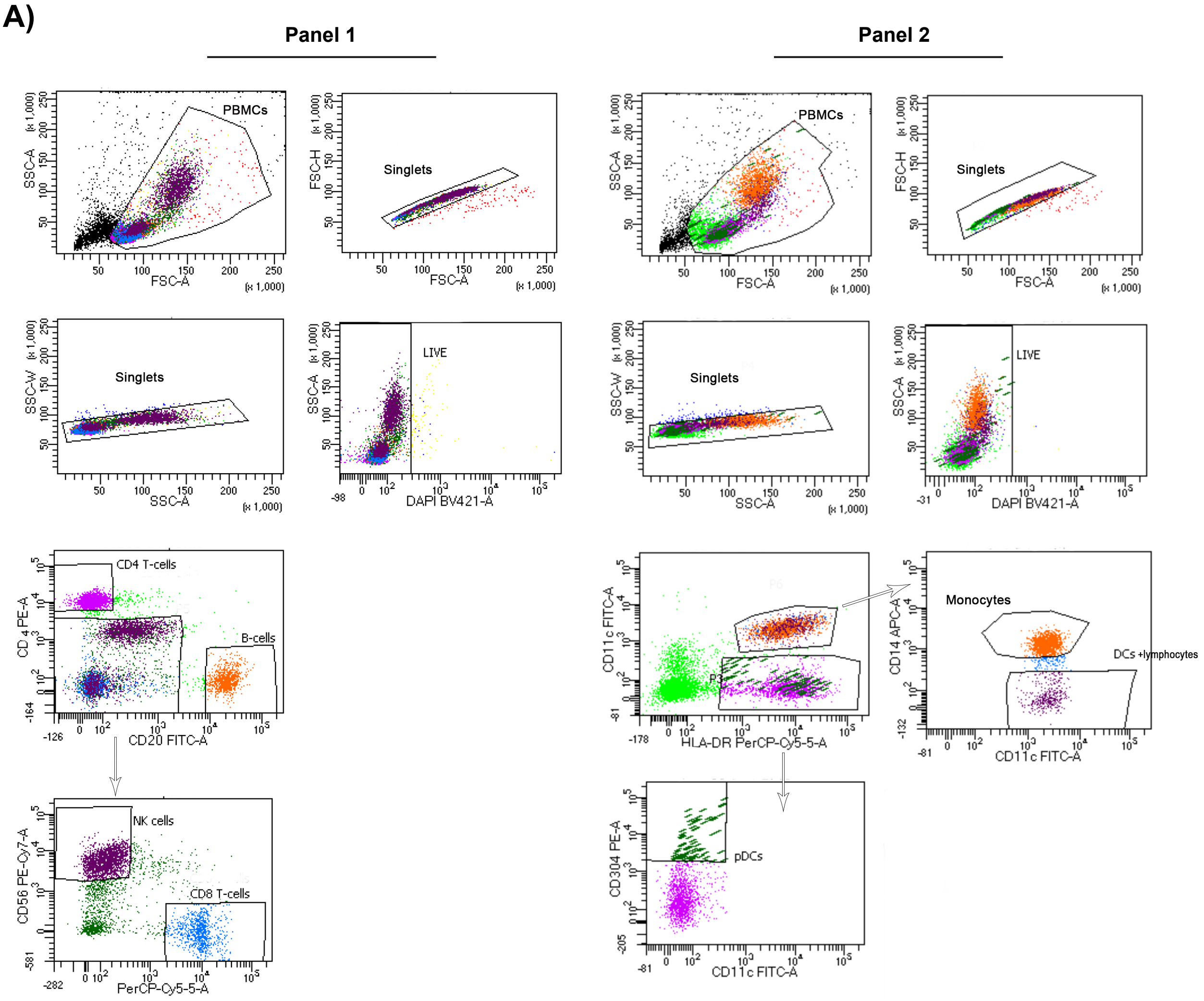

B)

Fluorochrome

Panels	FITC	PE	PerCP/Cyanine5.5	PE/Cyanine7	APC
1	CD20	CD4	CD8	CD56	-
2	CD11c	CD304	HLA-DR	-	CD14

Supplementary Figure S2 | Antibody markers and gating strategies in FACSorting of PBMC. **A**) Gating strategies to determine B cells, CD4^{+} T cells, CD8^{+} T cells, NK cells, monocytes, and pDCs. B cells are CD20^{+} CD4^{-}; CD4^{+} T cells are CD4^{+} CD20^{-}; CD8^{+} T cells are CD20^{-} CD4^{-}
CD8^{+}; NK cells are CD20^{-} CD3^{-} CD56^{+}; Monocytes are HLA-DR^{+} CD11c^{+} CD14^{+}; pDCs are HLA-DR^{+} CD11c^{-} CD304^{+}. **B**) Antibody marker panels used for cell sorting of PBMC.

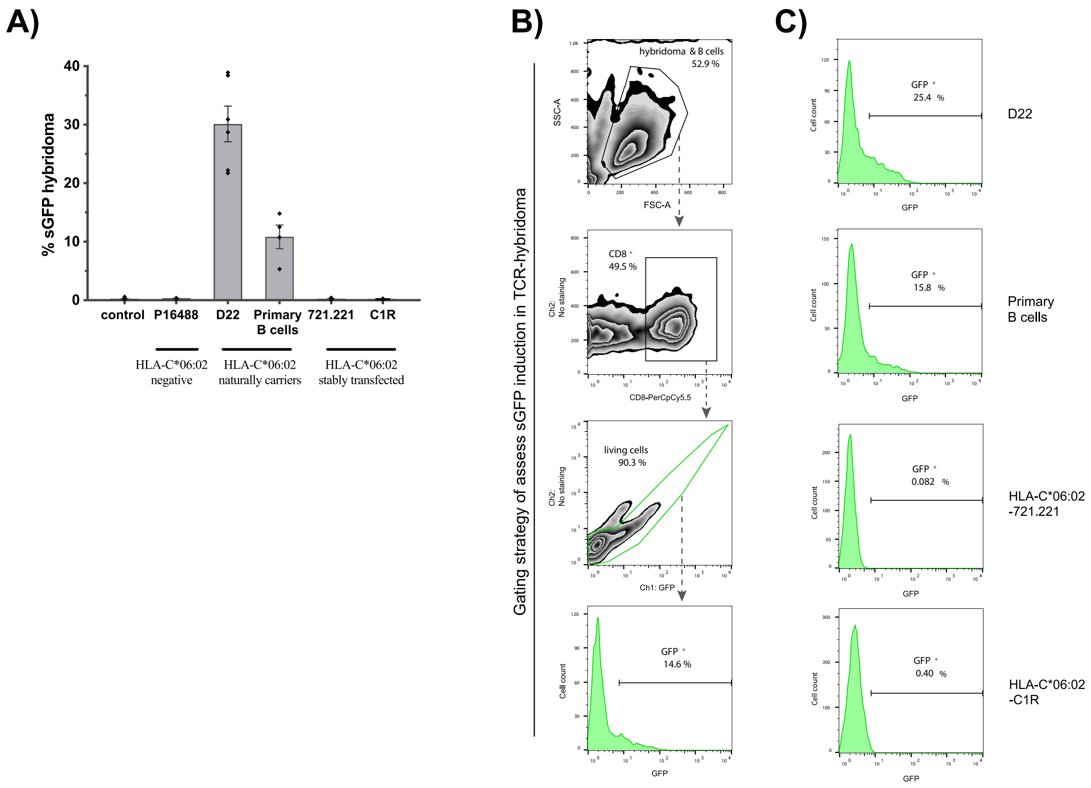

Supplementary Figure S3 | Analysis and gating strategy of Vα3S1/Vβ13S1-TCR hybridoma activation by the naturally *HLA-C*06:02*⁺ BCL D22, *HLA-C*06:02*⁺ primary B cells and the irradiation-modified HLA-C*06:02-transfected BCLs 721.221 and C1R. A) Activation of the Vα3S1/Vβ13S1-TCR hybridoma in co-culture with the HLA-C*06:02-transfected irradiati-on-modified BCLs (721.221 & C1R), as compared to other *HLA-C*06:02*⁺ (D22) or *HLA-C*06:02*⁻ (P16488) BCLs or *HLA-C*06:02*⁺ primary B cells. Data were assessed by multiparametric flow cytometry analysis and summarized from technical triplicates from
two or three independent experiments. **B)** Gating strategy to assess sGFP induction in TCR-hybridoma co-culture experiments. The Vα3S1/Vβ13S1-TCR hybridoma cells were differentiated from B cells or B cell lines by staining with PerCP/Cyanine5.5-conjuga-ted CD8 antibody, followed by the exclusion of TCR-hybridoma cells with high autofluorescence in Ch2. The GFP⁺ gate was set to
0.2% of GFP⁺ cells in non-stimulated hybridoma cells. **C)** Representative flow cytometry histograms of sGFP induction by the *HLA-C*06:02*⁺ BCL D22, primary B cells isolated from PBMC of a *HLA-C*06:02*⁺ psoriasis patient, and the irradiation-modified HLA-C*06:02-transfected BCLs 721.221 and C1R.

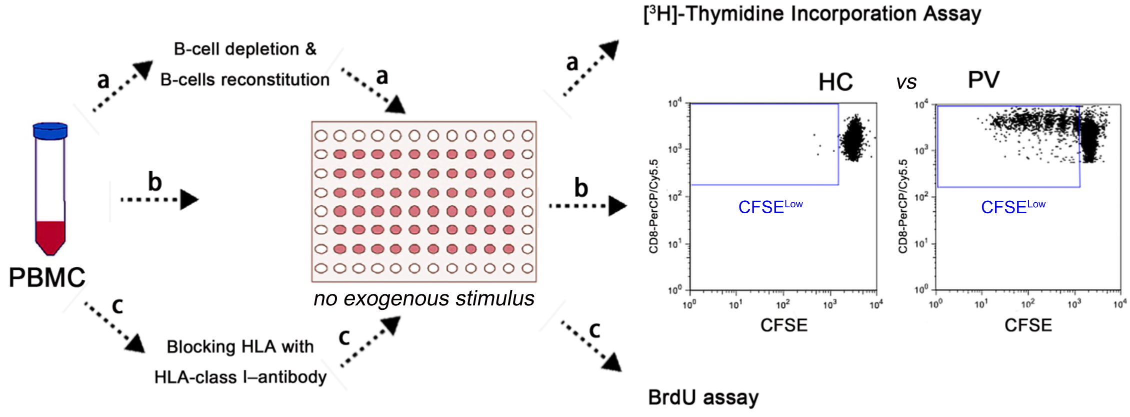

Supplementary Figure S4 | Workflow for assessing cell proliferation of PBMC *in vitro* in three sequential steps by different assays: **step a**, [^{3}H]-Thymidine incorporation assay; **step b**, CFSE-labeled assay to determine the number of generations of a cell by dilution of CFSE (CFSE^{low} population) that indicates proliferating cells, as shown here for CD8^{+} T cells;
step c, BrdU Cell Proliferation Assay. Cells were cultured in serum-free medium in the absence of exogenous stimulus for five days.

A)

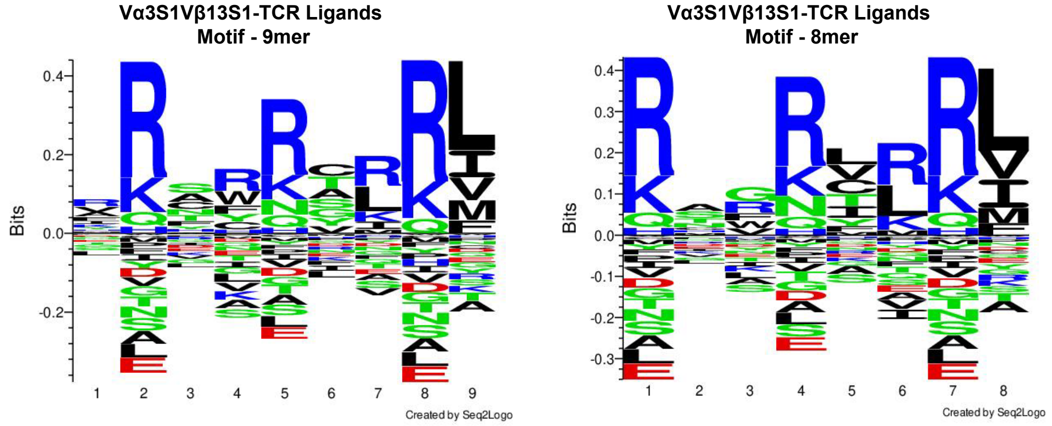

B)

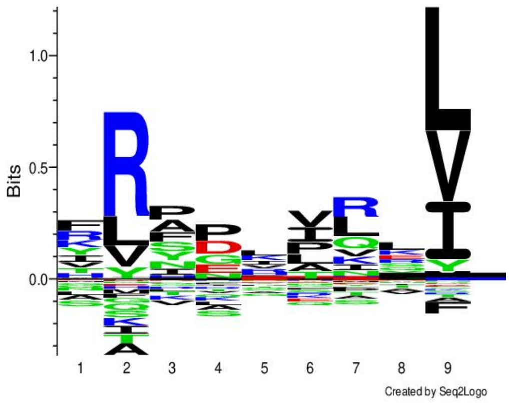

C)

	Motifs (encoded in PROSITE format)
1	x-[RY]-x(2)-[RNQTKHCILSV]-x-[RLSIV]-[RH]-[LFMVIF]
2	x-[RY]-x(2)-[RNQTKH]-x-[RLSIV]-[RH]-[LFMVIF]
3	x-[RY]-x(2)-[RNQTKH]-x-[RLSIV]-R-[LFMVIF]
4	x-[RY]-x(2)-[RNQTKH]-x-[RL]-R-[LMVIF]
5	x-[RY]-x(2)-[RNQ]-x-[RL]-R-[LMV]
6	x-R-x(2)-[RNQ]-x-[RL]-R-[LMV]
7	x-R-x(2)-R-x-[RL]-R-[LMV]
8	x-R-x(2)-R-x-[RL]-R-L
9	x-R-x(2)-R-x-R(2)-L

Supplementary Figure S5 | Sequence logos of the Vα3S1/Vβ13S1-TCR peptide recognition motifs and screening motifs and of the HLA-C*06:02 immunopeptidomes. A) 9-mer and 8-mer Vα3S1/Vβ13S1-TCR recognition motifs visualized employing Seq2Logo 2.0 with Kullback-Leibler logotype and default settings. The size of the letter indicates the impact of the corresponding amino acid, presented by a given position in either a positive or negative fashion. Amino acids are designated by the one-letter code. **B**) Peptide motif of the HLA-C*06:02 immunopeptidomes eluted from four *HLA-C*06:02*-homozygous B-cell lines predicted by NetMHCPan 4.0. **C**) Motifs used for screening against the HLA-C*06:02 immunopeptidomes from four human BCLs, in an order from strict to less strict. The motifs are encoded in PROSITE format.
